# Proteomics and metabolomics analyses of *Streptococcus agalactiae* isolates from human and animal sources

**DOI:** 10.1038/s41598-023-47976-y

**Published:** 2023-11-28

**Authors:** Shymaa Enany, Yasmine H. Tartor, Rania M. Kishk, Ahmed M. Gadallah, Eman Ahmed, Sameh Magdeldin

**Affiliations:** 1 Department of Microbiology and Immunology, Faculty of Pharmacy, Suez Canal University, Ismailia, 41522 Egypt; 2 Biomedical Research Department, Armed Force College of Medicine, Cairo, Egypt; 3 Department of Microbiology, Faculty of Veterinary Medicine, Zagazig University, Zagazig, 44511 Egypt; 4 Department of Medical Microbiology and Immunology, Faculty of Medicine, Suez Canal University, Ismailia, 41522 Egypt; 5 Department of Obstetrics and Gynecology, Faculty of Medicine, Suez Canal University, Ismailia, Egypt; 6 Proteomics and Metabolomics Unit, Department of Basic Research, Children’s Cancer Hospital Egypt 57357, Cairo, 11441 Egypt; 7 Department of Pharmacology, Faculty of Veterinary Medicine, Suez Canal University, Ismailia, 41522 Egypt; 8 Department of Physiology, Faculty of Veterinary Medicine, Suez Canal University, Ismailia, 41522 Egypt

**Keywords:** Microbiology, Proteomics

## Abstract

*Streptococcus agalactiae* (*S. agalactiae*), group B Streptococcus (GBS), a major cause of infection in a wide variety of diseases, have been compared in different human and animal sources. We aimed to compare the bacterial proteome and metabolome profiles of human and animal *S. agalactiae* strains to delineate biological interactions relevant to infection. With the innovative advancement in mass spectrometry, a comparative result between both strains provided a solid impression of different responses to the host. For instance, stress-related proteins (Asp23/Gls24 family envelope stress response protein and heat shock protein 70), which play a role in the survival of GBS under extreme environmental conditions or during treatment, are highly expressed in human and animal strains. One human strain contains ꞵ-lactamase (serine hydrolase) and biofilm regulatory protein (lytR), which are important virulence regulators and potential targets for the design of novel antimicrobials. Another human strain contains the aminoglycosides-resistance bifunctional AAC/APH (A0A0U2QMQ5) protein, which confers resistance to almost all clinically used aminoglycosides. Fifteen different metabolites were annotated between the two groups. L-aspartic acid, ureidopropionic acid, adenosine monophosphate, L-tryptophan, and guanosine monophosphate were annotated at higher levels in human strains. Butyric acid, fumaric acid, isoleucine, leucine, and hippuric acid have been found in both human and animal strains. Certain metabolites were uniquely expressed in animal strains, with fold changes greater than 2. For example, putrescine modulates biofilm formation. Overall, this study provides biological insights into the substantial possible bacterial response reflected in its macromolecular production, either at the proteomic or metabolomic level.

## Introduction

*Streptococcus agalactiae* (*S. agalactiae*), a group B Streptococcus (GBS), is a gram-positive bacterium that is considered an important pathogen regularly associated with mastitis in bovine and neonatal meningitis in humans. In humans, it is a dominant contributor to newborn infectious diseases in several regions, and a high number of females carry it asymptomatically. GBS is the leading cause of newborn sepsis and meningitis, with substantial mortality and morbidity, particularly in cases of extensive neonatal infections. It is also a significant pathogen in the elderly and in people with associated illnesses that impede immune defense systems against pathogenic microorganisms. Vertical GBS transmission to infants during accouchement is responsible for certain occurrences of neonatal GBS infections. Colonization occurs in approximately 40% of pregnant women. Approximately 5% of GBS-infected newborns die, while those who escape typically suffer from serious neurological consequences, such as mental retardation and sight or hearing impairment^1^.

In addition, *S. agalactiae* is considered one of the most frequent mastitis-causing bacteria in dairy cattle, causing significant reductions in the quantity and quality of milk and representing a huge economic problem for the dairy industry, particularly because this bacterium is extremely contagious within a farm and significantly affect milk productivity^2, 3^. GBS invades the mammary glands of dairy cattle through the skin and teats^4^. Mastitis caused by GBS is a chronic illness with few acute epidemics and no notable associated symptoms, but it affects milk output^5^.

The presence of *S. agalactiae* in humans and cattle raises the prospect of interspecies transmission. This concern is especially significant in view of the re-emergence of *S. agalactiae* in adults and animals. Numerous *S. agalactiae* comparisons between humans and cattle have been reported, and most studies have indicated that isolates from these species constitute substantially separate groups in terms of their core and accessory genomes^6–8^.

There is barely any information at the level of the functional proteome presented in GBS and its physiological status in both human and bovine GBS. Proteomics reveals valuable information about the virulence of the pathogen and is a very useful procedure for understanding how bacteria respond to external environmental factors. It is a powerful method to investigate bacterial response profiling from system-level studies and can enhance our knowledge of bacterial adaptation, antibiotic resistance mechanisms, and tolerance development^9^. This aids in a deeper understanding of infection and the advancement of antimicrobial strategies to fight infection.

On the other side, metabolomics is a comprehensive examination of the metabolites found within the living systems. The approach has been widely used to help better understand the complex bacterial cellular metabolic states and changes as one of the important "omics" tools. It has also provided important information for elucidating the disease pathophysiology and identifying novel biomarkers^10^. The availability of databases on bacterial gene regulation and metabolic pathways allows for the investigation of prevalent bacterial systems, such as strain identification and differentiation, drug mechanisms of action, and metabolic changes in response to antimicrobial treatment^11^.

To the best of our knowledge, no published data have investigated the link between the proteomic profile of clinical human and animal *S. agalactiae* strains and their related metabolites. In this study, we used different techniques to compare the proteomic and metabolomic signatures of human and animal *S. agalactiae* strains.

## Results

### Antimicrobial resistance profiles

All the GBS strains (100%) were resistant to penicillin, amoxicillin, clindamycin, ceftriaxone, cephalexin, cloxicillin, cefepime, tetracycline, erythromycin, and streptomycin followed by ciprofloxacin (69.6%), amoxicillin-clavulanic acid (60.9%), and cefoperazone (43.5%). However, high susceptibility was observed for impenem (100%) followed by sulfamethaxole-trimethoprime (69.6%). All isolates were multidrug-resistant (MDR) with multiple antibiotic resistant index (MAR) ranged from 0.66 to 0.93 (Supplementary Table 1).

### Proteomic analysis

#### Comprehensive proteome analysis of *S. agalactiae* strains

Brain heart infusion (BHI) broth sub-culture of *S. agalactiae* for both human and animal strains was used for protein extraction. The proteomes of 23 samples (16 of human origin and 7 of animal origin) were analyzed. Quality validation showed that distribution of mass errors was lower than 0.02 Da indicating a good quality of sample preparation. A total of 2527 *S. agalactiae* proteins were identified with 99% confidence at the peptide and protein levels (Supplementary Table 2). The total number of proteins identified in *S. agalactiae* strains of human origin and in those of animal origins were 1271 and 929, respectively. Refinement based on repeated gene names resulted in 1090 and 859 proteins for human and animal strains, respectively (Supplementary Tables 3 and 4).

Of the annotated components, 1659/2527 have an assigned molecular function, 1082/2527 are involved in cellular compartment, and 1080/2527 are assigned known biological processes (Supplementary Table 2).

The identified proteins are available in the PRIDE database (www.ebi.ac.uk/pride) via ProteomeXchange with identifier PXD035678.

#### Proteome profile of human and animal strains

The total area under the peak was used to normalize the samples. Box plots and kernel density plots before and after normalization are displayed in Supplementary Fig. 1.

**Figure 1:**
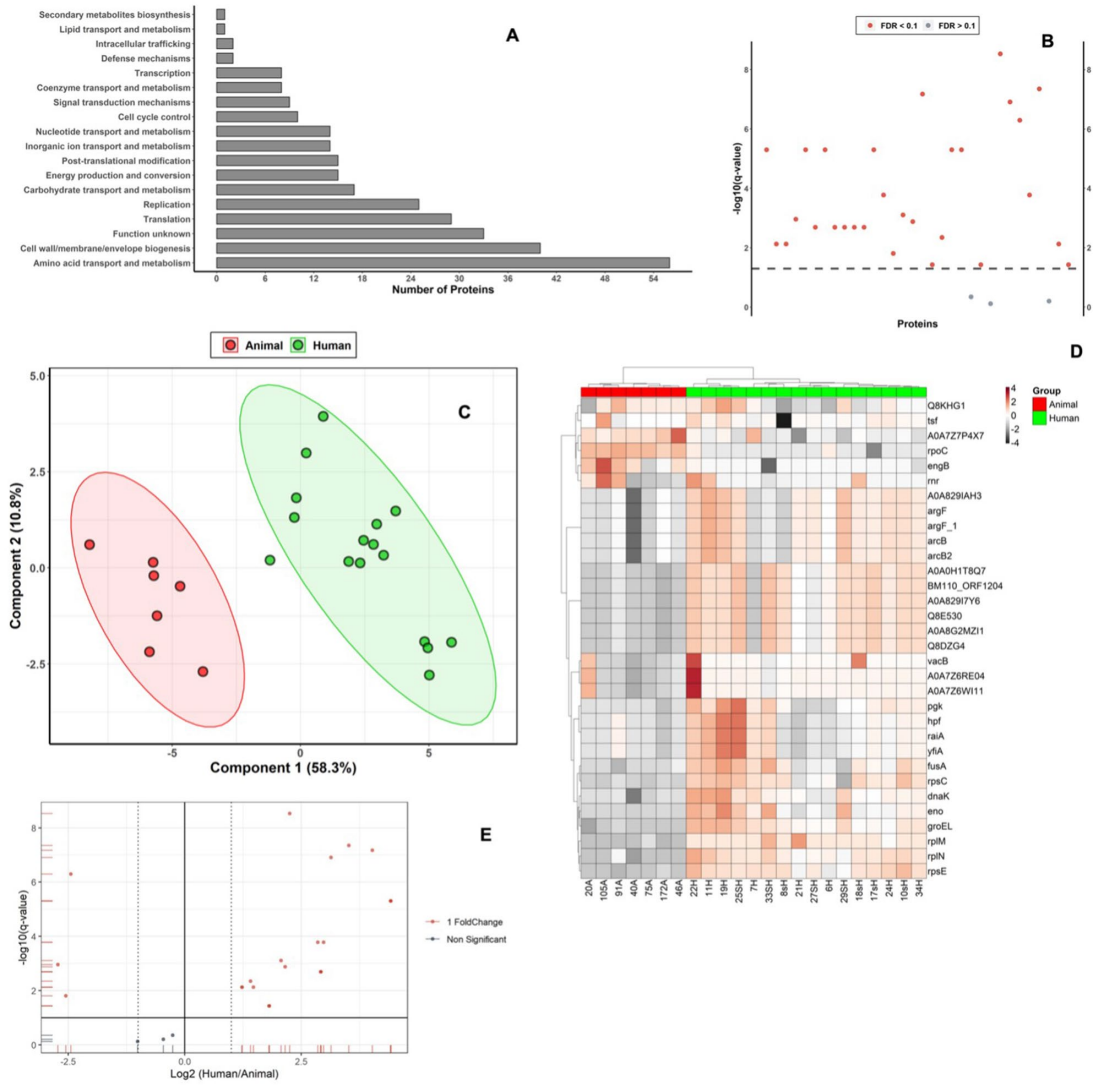
(**A**) Most abundant protein classes identified in human *S. agalactiae* strains, x axis shows number of proteins involved in the pathway, Y axis shows KEGG pathway. This figure was created using the KEGG database developed by Kanehisa Laboratories (www.kegg.jp/kegg/kegg1.html). (**B**) T-test with a *p*-value of 0.05 and FDR = 1% between proteins in human and animals *S. agalactiae* strains**,** red dots represent significant protein between both groups. (**C**) Partial least squares—discriminant analysis (PLS-DA) showing differences in proteome profile between both experimental groups. Both first and second components discriminate human and animals *S. agalactiae* proteome strains by 69.1%. (**D**) Clustering heatmap showing protein abundance of human and animals *S. agalactiae* strains. X axis represents samples with clear separation in proteome abundances of both strains**.** The Y axis represents gene symbol of protein. Scale represents scaled data. Heatmap is generated using the ggplot 2 package for the statistical programming language R (Version 3.4.3, https://ggplot2.tidyverse.org). (**E**) Volcano plot showing significantly dysregulated proteins between both strains. X axis represents log2 fold change differences of human/ animal where onefold change differences was set as a cutoff value. Y axis represents − log 10 of q value.

The most abundant protein classes identified in human strains were amino acid transport and metabolism, cell wall/membrane/envelop biogenesis, unknown function, translation, and replication proteins (Fig. 1A).

We found that the abundances of 32 proteins had changed significantly using Mann Whitney test with a *p*-value of 0.05, FDR 1%, and a fold change cutoff point of 2 (Fig. 1B). In Fig. 1C, principal component analysis (PCA) showed significant differences between the host proteomes evidenced by distinct patterns of variance (96.1% for the first 2 components) captured by the principal components, indicating a clear separation in the multivariate data distribution of the groups. The clustering heatmap between these different hits showed unique proteins with high abundance for human and animal strains (Fig. 1D).

All the differentially accumulated proteins (DAPs) were annotated to a specific function using the Gene Ontology (GO) and Kyoto Encyclopedia of Genes and Genomes (KEGG) databases.

GO annotation indicated that the DAPs of human and animal strains could be classified into 18 and 16 categories, respectively, which included many important biological and cellular processes (Supplementary Tables 2, 5, and 6, Supplementary Fig. 2A and 2B).

The most assigned functional categories in human strains were amino acid transport and metabolism (18.73%), cell wall/membrane/envelop biogenesis (13.38%), unknown function (11.04%), translation (9.69%), and replication (8.36%). The other protein functions were carbohydrate transport and metabolism, energy production and conversion, post-translational modification, inorganic ion transport and metabolism, nucleotide transport and metabolism, cell cycle control, signal transduction mechanisms, coenzyme transport and metabolism, transcription, defence mechanisms, intracellular trafficking, lipid transport and metabolism, and secondary metabolites biosynthesis.

Concerning animal strains, most of the annotated proteins functions were replication (20.3%), carbohydrate transport and metabolism (16.51%), amino acid transport and metabolism (9.91%), signal transduction mechanisms (8.49%), post-translational modification (8.02%), and defense mechanisms (6.13%). KEGG orthology (KO) annotation for these proteins revealed that the preponderant reported KO for unique proteins of both human and animals *S. agalactiae* strains were involved in microbial metabolism, genetic information processing, and defense mechanism (Supplementary Figs. 3 and 4).

#### Unique and shared proteins between human and animals *S. agalactiae* strains

**Figure 2:**
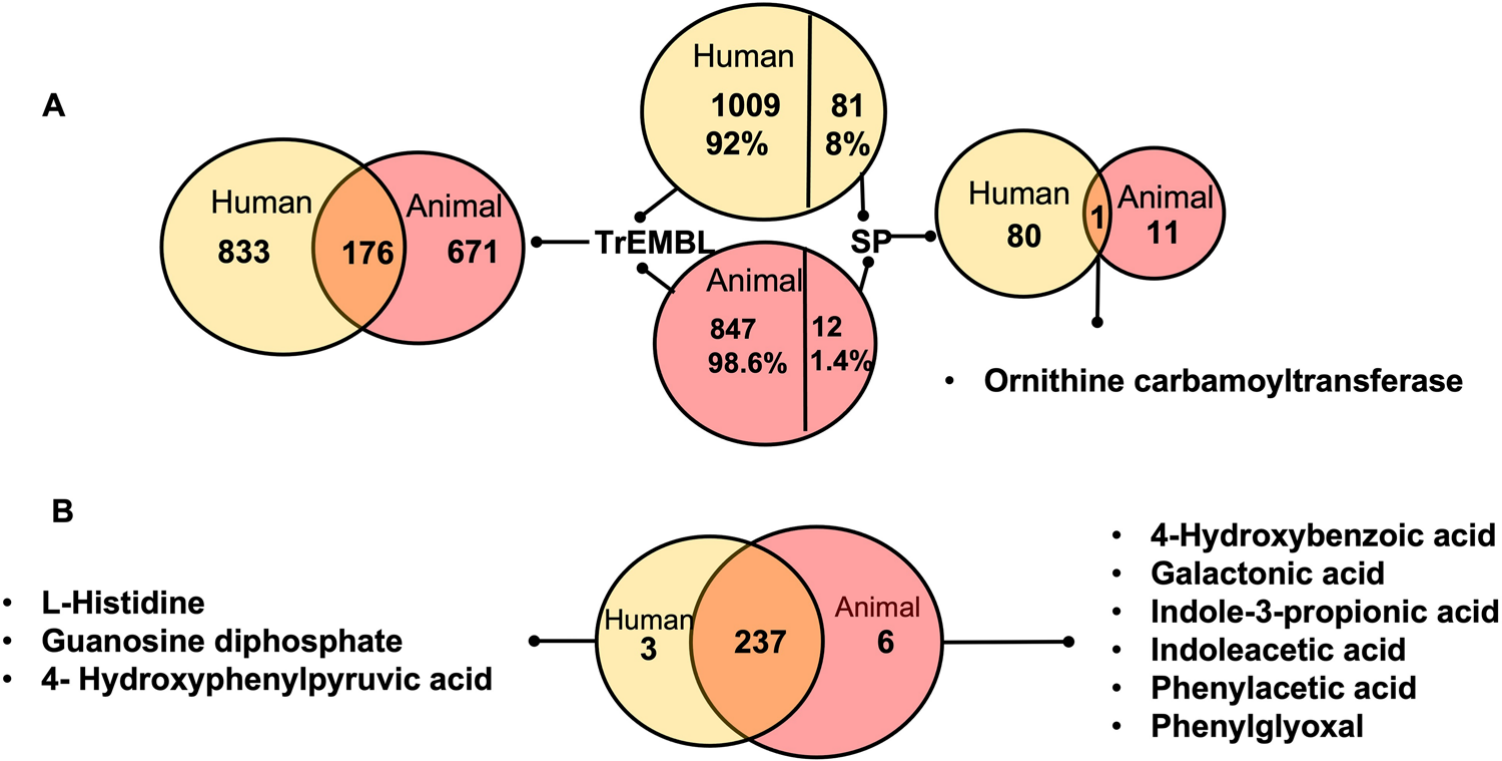
(**A**) Proteome comparison between human and animals *S. agalactiae* strains. Number of proteins identified in human strains were 1090; from them 1009 identified by TrEMBL and 81 by SwissProt, while the number of proteins identified in animal strains were 859; from them 847 identified by Tremble and 12 by SwissProt. Shared proteins between the two groups were 176 by TrEMBL and 1 by SwissProt. (**B**) Metabolome comparison between human and animals *S. agalactiae* strains. Number of metabolites identified were 240 and 243 in human and animal strains; respectively, from them 237 were shared.

Proteome difference between human and animals *S. agalactiae* strains was clearly observed (Fig. 2A). Both proteomes yielded 1090 and 859 proteins for human and animal strains, respectively. As expected, most of them were non curated TrEMBL with 176 sharing. On the other hand, curated proteins identified by Swiss-prot (SP) were only 81 and 12 in human and animal strains; respectively and mostly different. Only 1 shared protein between human and animal was found by SP: Catabolic Ornithine Carbamoyltransferase. Detailed proteome information is found in Supplementary Tables 3 and 4.

Differentially shared proteins between human and animals *S. agalactiae* strains were plotted by volcano plot with the magnitude of difference in expression value (Log2) and *P* value between average biological replicates of the two groups (Fig. 1E). The 29 significantly shared proteins between the two groups identified by volcano plot were listed in Table 1.

**Table 1:** Differentially significant shared proteins between human and animals *S. agalactiae* strains with annotated gene ontology.

Protein name	Gene	Accession number	FC	log2(FC)	p. adjusted	− log10 (p)	Biological Function	Cellular component	Molecular Function
50S ribosomal protein L13	*rpl*M	A0A0E1EFE5	4.7632	2.2519	2.95E−09	8.53	Translation	Cytoplasm; ribosome	Structural constituent of ribosome
30S ribosomal protein S5	*rps*E	Q3K3V2	11.47	3.5198	4.49E−08	7.35	Translation	Small ribosomal subunit	rRNA binding ; structural constituent of ribosome
Chaperonin GroEL (EC 5.6.1.7) (60 kDa chaperonin)	*gro*EL	Q3JYQ0	16.271	4.0242	6.67E−08	7.18	Protein refolding	Cytoplasm	ATP binding ; ATP hydrolysis activity; isomerase activity ; unfolded protein binding
50S ribosomal protein L14	*rpl*N	V6YZ40	8.7988	3.1373	1.24E−07	6.91	Translation	Cytoplasm; large ribosomal subunit	rRNA binding ; structural constituent of ribosome
DNA-directed RNA polymerase subunit beta′ (RNAP subunit beta′)	*rpo*C	Q3K3L1	0.18381	− 2.4437	5.11E−07	6.29	Transcription, DNA-templated		DNA binding; DNA-directed 5′-3′ RNA polymerase activity; magnesium ion binding; zinc ion binding
Asp23/Gls24 family envelope stress response protein	A0A8G2MZI1	A0A8G2MZI1	21.435	4.4219	5.02E−06	5.3			
Gls24 protein, putative	Q8DZG4	Q8DZG4	21.414	4.4205	5.02E−06	5.3			
Asp23/Gls24 family envelope stress response protein (Stress response regulator gls24-like protein)	A0A0H1T8Q7	A0A0H1T8Q7	21.399	4.4195	5.02E−06	5.3			
Uncharacterized protein	Q8E530	Q8E530	21.377	4.418	5.02E−06	5.3			
Asp23/Gls24 family envelope stress response protein (General stress protein, Gls24 family) (Stress response regulator Gls24)	BM110_ORF1204	I7ISQ2	21.365	4.4172	5.02E−06	5.3			
Stress response regulator Gls24	A0A829I7Y6	A0A829I7Y6	21.351	4.4163	5.02E−06	5.3			
30S ribosomal protein S3	*rps*C	P66558	7.8646	2.9754	0.000167	3.78	Translation	Ribosome	mRNA binding; rRNA binding; structural constituent of ribosome
Chaperone protein DnaK (HSP70) (Heat shock 70 kDa protein) (Heat shock protein 70)	*dna*K	P0A3J2	7.2312	2.8542	0.000167	3.78	Protein folding		ATP binding ; ATP hydrolysis activity; unfolded protein binding
Enolase (EC 4.2.1.11) (2-phospho-D-glycerate hydro-lyase) (2-phosphoglycerate dehydratase)	*eno*	P64080	4.1835	2.0647	0.000783	3.11	Glycolytic process	Cell surface; extracellular region; phosphopyruvate hydratase complex	Magnesium ion binding ; phosphopyruvate hydratase activity
Uncharacterized protein	A0A7Z7P4X7	A0A7Z7P4X7	0.15127	− 2.7248	0.001098	2.96		Integral component of membrane	
Elongation factor G (EF-G)	*fus*A	Q3JZB5	4.4542	2.1552	0.001325	2.88			GTPase activity ; GTP binding ; translation elongation factor activity
Ornithine carbamoyltransferase (OTCase) (EC 2.1.3.3)	A0A829IAH3	A0A829IAH3	7.5705	2.9204	0.002039	2.69			Transferase activity
Ornithine carbamoyltransferase (OTCase) (EC 2.1.3.3)	*arg*F	A0A380IHZ9	7.566	2.9195	0.002039	2.69		Cytoplasm	Amino acid binding ; ornithine carbamoyltransferase activity
Ornithine carbamoyltransferase (OTCase) (EC 2.1.3.3)	*arg*F_1	A0A076YXU9	7.5648	2.9193	0.002039	2.69		Cytoplasm	Amino acid binding ; ornithine carbamoyltransferase activity
Ornithine carbamoyltransferase 2, catabolic (OTCase 2) (EC 2.1.3.3)	*arc*B2	P65605	7.5584	2.9181	0.002039	2.69	Arginine catabolic process to ornithine; arginine deiminase pathway	Cytoplasm	Amino acid binding; ornithine carbamoyltransferase activity
Ornithine carbamoyltransferase, catabolic (OTCase) (EC 2.1.3.3)	*arc*B	Q8RP83	7.5577	2.918	0.002039	2.69	Arginine catabolic process to ornithine	Cytoplasm	Amino acid binding; ornithine carbamoyltransferase activity
Phosphoglycerate kinase (EC 2.7.2.3)	*pgk*	Q3JZB8	2.66	1.4114	0.004486	2.35	Glycolytic process	Cytoplasm	ATP binding; phosphoglycerate kinase activity
Ribonuclease R (RNase R) (EC 3.1.13.1)	*vac*B	A0A0H1VU98	2.7754	1.4727	0.007517	2.12		Cytoplasm	Exoribonuclease II activity; RNA binding
Ribonuclease R (Fragment)	A0A7Z6WI11	A0A7Z6WI11	2.3472	1.2309	0.007517	2.12			Ribonuclease activity; RNA binding
Ribonuclease R (Fragment)	A0A7Z6RE04	A0A7Z6RE04	2.3416	1.2275	0.007517	2.12			Ribonuclease activity; RNA binding
Probable GTP-binding protein EngB	*eng*B	V6YZA7	0.17017	− 2.5549	0.01563	1.81	Division septum assembly		GTP binding; metal ion binding
Ribosome hibernation promoting factor (HPF)	*hpf*	A0A806NFT9	3.5157	1.8138	0.037395	1.43			
Ribosome hibernation promoting factor (HPF)	*yfi*A	A0A0H1YFL3	3.5099	1.8114	0.037395	1.43	Primary metabolic process; regulation of translation	Cytoplasm	
Ribosome hibernation promoting factor (HPF)	*rai*A	A0A0E1EL02	3.5065	1.81	0.037395	1.43	Primary metabolic process; regulation of translation	Cytoplasam	

*FC* fold change (human/animal). All data retrieved from UniProt.

The KEGG orthology annotation for these shared proteins identified proteins associated with pathogenesis (two-component system and RNA degradation), Glycolysis/Gluconeogenesis, and RNA polymerase (Table 2). These proteins were significantly upregulated in human samples with a fold change magnitude ranged from 2.34 to 21.435. However, DNA-directed RNA polymerase subunit beta, probable GTP-binding protein EngB, and uncharacterized protein A0A7Z7P4X7 were downregulated (Table 1 and Supplementary Fig. 5).

**Table 2:** KEGG orthology of shared proteins between human and animals *S. agalactiae* strains.

Name	Description	Accession number	Protein name	Gene name	KEGG Orthology (KO)	Locus-tag
RNA degradation	ko03018	P0A3J2	Chaperone protein DnaK (HSP70) (Heat shock 70 kDa protein) (Heat shock protein 70)	*dna*K	03,018 RNA degradation [PATH:ko03018]	sp|P0A3J2|DNAK_STRA3
Glycolysis/gluconeogenesis	ko00010	P64080	Enolase (EC 4.2.1.11) (2-phospho-D-glycerate hydro-lyase) (2-phosphoglycerate dehydratase)	*eno*	00010 Glycolysis/Gluconeogenesis [PATH:ko00010]	sp|P64080|ENO_STRA3
Ribosome	ko03010	P66558	30S ribosomal protein S3	*rpsC*	03,010 Ribosome [PATH:ko03010]	sp|P66558|RS3_STRA3
Glycolysis/Gluconeogenesis	ko00010	Q3JZB8	Phosphoglycerate kinase (EC 2.7.2.3)	*pgk*	00010 Glycolysis/Gluconeogenesis [PATH:ko00010]	sp|Q3JZB8|PGK_STRA1
RNA polymerase	ko03020	Q3K3L1	DNA-directed RNA polymerase subunit beta' (RNAP subunit beta') (EC 2.7.7.6) (RNA polymerase subunit beta') (Transcriptase subunit beta')	*rpo*C	03,020 RNA polymerase [PATH:ko03020]	sp|Q3K3L1|RPOC_STRA1
Ribosome	ko03010	Q3K3V2	30S ribosomal protein S5	*rps*E	03,010 Ribosome [PATH:ko03010]	sp|Q3K3V2|RS5_STRA1
Two-component system	ko02020	Q8RP83	Ornithine carbamoyltransferase, catabolic (OTCase) (EC 2.1.3.3)	*arc*B	02,020 Two-component system [PATH:ko02020]	sp|Q8RP83|OTCC_STRAG
Two-component system	ko02020	A0A076YXU9	Ornithine carbamoyltransferase (OTCase) (EC 2.1.3.3)	*arg*F_1	02,020 Two-component system [PATH:ko02020]	tr|A0A076YXU9|A0A076YXU9_STRAG
Ribosome	ko03010	A0A0E1EFE5	50S ribosomal protein L13	*rpl*M	03,010 Ribosome [PATH:ko03010]	tr|A0A0E1EFE5|A0A0E1EFE5_STRAG
Two-component system	ko02020	A0A380IHZ9	Ornithine carbamoyltransferase (OTCase) (EC 2.1.3.3)	*arg*F	02,020 Two-component system [PATH:ko02020]	tr|A0A380IHZ9|A0A380IHZ9_STRAG
Two-component system	ko02020	A0A829IAH3	Ornithine carbamoyltransferase (OTCase) (EC 2.1.3.3)	A0A829IAH3	02,020 Two-component system [PATH:ko02020]	tr|A0A829IAH3|A0A829IAH3_STRAG
Ribosome	ko03010	V6YZ40	50S ribosomal protein L14	*rpl*N	03,010 Ribosome [PATH:ko03010]	tr|V6YZ40|V6YZ40_STRAG

### Metabolomics analysis

#### Identification and comparison of metabolites from human and animal *S. agalactiae* strains

BHI broth sub-culture of *S. agalactiae* for both human and animal strains was used for metabolites extraction. The metabolome of 23 strains (16 of human origin and 7 of animal origin) were analyzed. A total of 325 measurable and reproducible metabolite signals were obtained from both human and animal strains (Supplementary Table 7A). After applying a filtration criterion to require at least 50% existence of the metabolite/group, a total of 246 metabolites were retrieved (Supplementary Table 7B). Notably, 237 were shared and 9 were unique metabolites (Supplementary Table 7B).

Metabolome difference between human and animals *S. agalactiae* strains revealed 3 and 6 unique metabolites in human and animal strains, respectively (Fig. 2B). L-histidine, guanosine diphosphate, and hydroxyphenylpyruvic acid (HPPA) were uniquely found in human strains, while 4-hydroxybenzoic acid, galactonic acid, indole-3-propionic acid, indoleacetic acid, phenylacetic acid, and phenylglyoxal were uniquely identified in animal strains. Using the HMDB metabolomics library, 241 annotated metabolites consisted mainly of amino acids, carbohydrates, peptides, organic acids, fatty acids, and pyrimidines were annotated after strict quality control (Supplementary Table 7C).

PCA^12^ and OPLS-DA^13^ were used to describe the differences between the two groups' metabolic profiles. *S. agalactiae* strains from the human group and those from the animal group could be easily distinguished from one another, according to the PCA score plots (Supplementary Fig. 6A). 40.8 and 11.3% of the variance, respectively, were represented by principal components 1 and 2. The OPLS-DA findings revealed that the metabolite compositions of the two groups were unique and significantly different (Supplementary Fig. 6B). Validation plots might be used to depict the parameters for evaluating the OPLS-DA model's quality (Supplementary Fig. 7).

**Figure 3:**
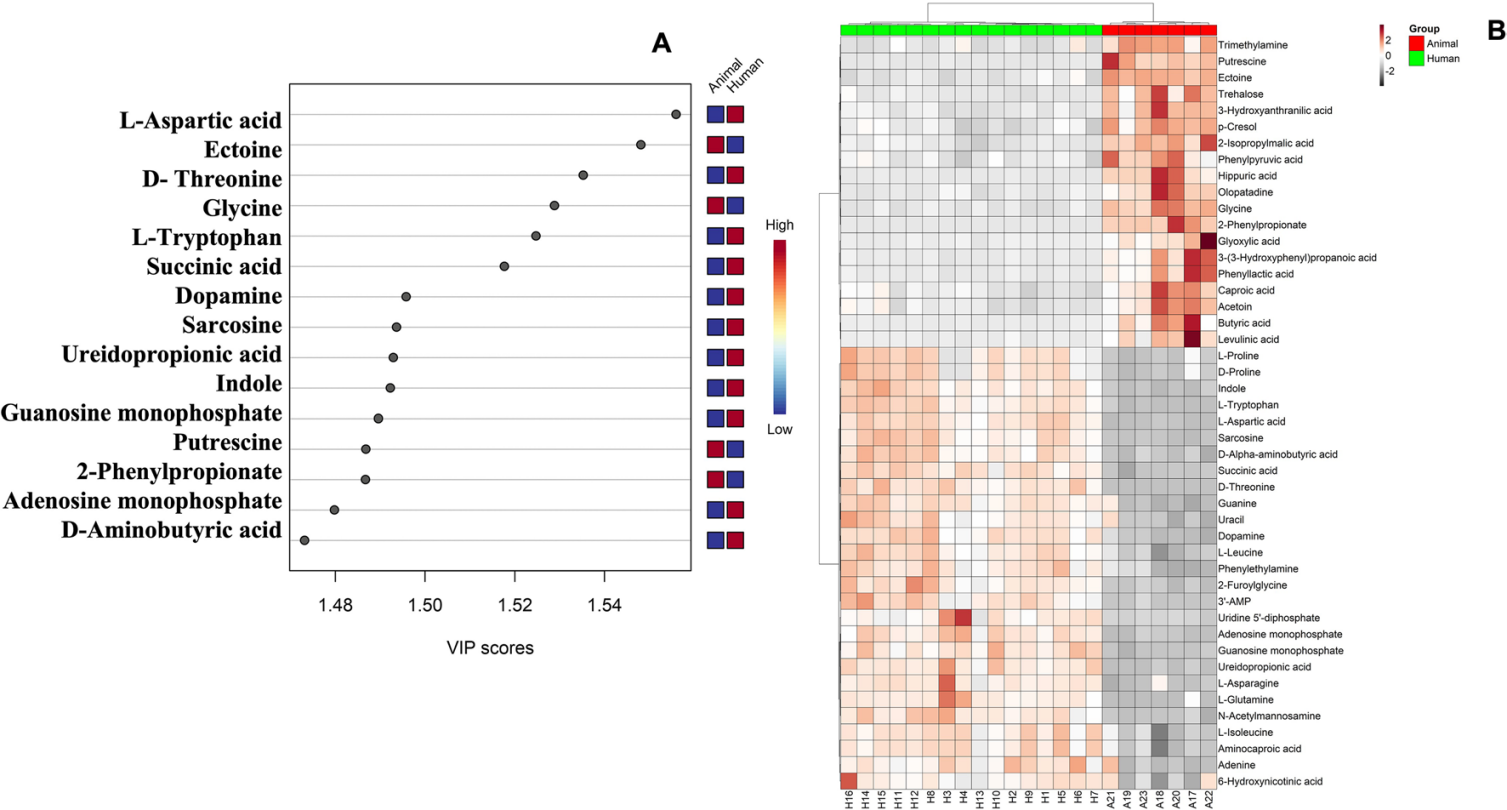
(**A**) Variable importance in projection (VIP) scores obtained from the OPLS-DA showing top 15 discriminating metabolites between human and animal *S. agalactiae* strains, (**B**) Metabolome heatmap showing differences in the metabolites abundance (scaled) annotated between human and animal *S. agalactiae* strains. X axis represents samples and Y axis represents metabolite. Heatmap is generated using the ggplot 2 package for the statistical programming language R (Version 3.4.3, https://ggplot2.tidyverse.org).

As presented in Fig. 3A, VIP scores obtained from the OPLS-DA (*p* < 0.05 and VIP > 1) indicated significant increases in the relative concentration of 15 different metabolites/small molecules between the two groups. The different metabolites included L-aspartic acid, glycine, L-tryptophan, succinic acid, dopamine, sarcosine, ureidopropionic acid, indole, guanosine monophosphate, putrescine, 2-phenylpropionate, adenosine monophosphate, and D-alpha-aminobutyric acid.

Human strains contained higher levels of L-aspartic acid, ureidopropionic acid, adenosine monophosphate, L-tryptophan, and guanosine monophosphate than did animal strains.

### Metabolic pathways enrichment analysis

To better evaluate how multiple pathways differed between the human and the animal strains, a KEGG functional enrichment analysis of the pathways related to the different metabolites was conducted (Table 3).

**Table 3:** Differentially significant metabolites with their KEGG pathways in human over animal *S. agalactiae* strains.

Up/down	KEGG identifier	Annotation	Category	N	n	M	m	*p*-value	FDR correction
Down	sag00660	C5-Branched dibasic acid metabolism	Pathways (*S. agalactiae* 2603 (serotype V))	28	5	3150	32	6.51E−06	0.000234431
Down	sag00360	Phenylalanine metabolism	Pathways (*S. agalactiae* 2603 (serotype V))	28	5	3150	46	4.07E−05	0.000732241
Down	sag00290	Valine, leucine and isoleucine biosynthesis	Pathways (*S. agalactiae* 2603 (serotype V))	28	4	3150	28	8.84E−05	0.001060756
Down	sag00650	Butanoate metabolism	Pathways (*S. agalactiae* 2603 (serotype V))	28	4	3150	40	0.000366611	0.003299495
Down	sag00480	Glutathione metabolism	Pathways (*S. agalactiae* 2603 (serotype V))	28	3	3150	38	0.004310425	0.031035062
Down	sag00630	Glyoxylate and dicarboxylate metabolism	Pathways (*S. agalactiae* 2603 (serotype V))	28	3	3150	44	0.006530197	0.039181183
Down	sag00260	Glycine, serine and threonine metabolism	Pathways (*S. agalactiae* 2603 (serotype V))	28	3	3150	49	0.008818752	0.04535358
Down	sag00020	Citrate cycle (TCA cycle)	Pathways (*S. agalactiae* 2603 (serotype V))	28	2	3150	20	0.01311596	0.059021818
Down	sag00643	Styrene degradation	Pathways (*S. agalactiae* 2603 (serotype V))	28	2	3150	27	0.023320296	0.093281185
Up	sag00970	Aminoacyl-tRNA biosynthesis	Pathways (*S. agalactiae* 2603 (serotype V))	33	9	3150	75	3.68E−08	1.51E-06
Up	sag01100	Metabolic pathways	Pathways (*S. agalactiae* 2603 (serotype V))	33	29	3150	1455	6.41E−07	1.31E-05
Up	sag00230	Purine metabolism	Pathways (*S. agalactiae* 2603 (serotype V))	33	8	3150	92	2.97E−06	3.48E-05
Up	sag00250	Alanine, aspartate and glutamate metabolism	Pathways (*S. agalactiae* 2603 (serotype V))	33	5	3150	24	3.40E−06	3.48E-05
Up	sag02010	ABC transporters	Pathways (*S. agalactiae* 2603 (serotype V))	33	7	3150	90	2.87E−05	0.000235176
Up	sag00910	Nitrogen metabolism	Pathways (*S. agalactiae* 2603 (serotype V))	33	4	3150	26	0.000126967	0.000867608
Up	sag00330	Arginine and proline metabolism	Pathways (*S. agalactiae* 2603 (serotype V))	33	6	3150	82	0.000163401	0.000957064
Up	sag00770	Pantothenate and CoA biosynthesis	Pathways (*S. agalactiae* 2603 (serotype V))	33	3	3150	27	0.002582544	0.011764924
Up	sag00400	Phenylalanine, tyrosine and tryptophan biosynthesis	Pathways (*S. agalactiae* 2603 (serotype V))	33	3	3150	27	0.002582544	0.011764924
Up	sag00240	Pyrimidine metabolism	Pathways (*S. agalactiae* 2603 (serotype V))	33	4	3150	59	0.003029335	0.01185458
Up	sag02020	Two-component system	Pathways (*S. agalactiae* 2603 (serotype V))	33	3	3150	29	0.003180497	0.01185458
Up	sag00471	D-Glutamine and D-glutamate metabolism	Pathways (*S. agalactiae* 2603 (serotype V))	33	2	3150	12	0.006579544	0.022480109
Up	sag00760	Nicotinate and nicotinamide metabolism	Pathways (*S. agalactiae* 2603 (serotype V))	33	3	3150	44	0.010360598	0.032675733
Up	sag00260	Glycine, serine and threonine metabolism	Pathways (*S. agalactiae* 2603 (serotype V))	33	3	3150	49	0.013910373	0.04073752
Up	sag00290	Valine, leucine and isoleucine biosynthesis	Pathways (*S. agalactiae* 2603 (serotype V))	33	2	3150	28	0.033951687	0.092801279

*N* Total metabolites in the pathway, *n* The number of differentially significant metabolites identified in the cohorts between the 2 groups, *M* Total number of metabolites being considered in our analysis, *m* Number of differentially significant metabolites, *FDR* false discovery rate.

Pathway topology analysis showed 29 shared metabolic pathways between the two groups, mainly metabolic pathway, phenylalanine metabolism, ABC transporters, alanine, aspartate and glutamate metabolism, pyrimidine metabolism, butanoate metabolism, glycine, serine and threonine metabolism, phenylalanine, tyrosine and tryptophan biosynthesis, arginine and proline metabolism, purine metabolism, two-component system, aminoacyl-tRNA biosynthesis, D-glutamine and D-glutamate metabolism, and citrate cycle (TCA cycle) (Supplementary Fig. 8).

The unique metabolites in human group (n = 16) included L-histidine, L-arginine, guanosine diphosphate, isopentenyl pyrophosphate, malonic semialdehyde, alpha-linolenic acid, guanosine, 2-phenylbutyric acid, pargyline, 1-pyrroline, O-phosphoethanolamine, phosphorylcholine, 4-hydroxyphenylpyruvic acid, amphetamine, 2,6-dimethylpyridine, and PE (16:1(9Z)/16:1(9Z)). Whereas, 12 metabolites were uniquely annotated in animal group including 3-methyl pyruvic acid, galactonic acid, indole-3-propionic acid, ꞵ-hydroxy butyric acid, indoleacetic acid, phenylacetic acid, rivastigmine, deoxyuridine, oxalic acid, phenylglyoxal, 3-(Methylthio)-1-propene, and niacinamide.

As revealed in Fig. 3B, the metabolome heatmap indicated the differences between the two groups. Certain metabolites were uniquely expressed in animal strains with fold change magnitude exceeding 2. For instances, putrescine which plays a role in modulation of biofilm formation and disassembly and consequently raising the colonization of the bacteria. Also, phenylacetic acid which is a central intermediate metabolite involved in bacterial degradation of aromatic components that are involved in biofilm formation and antimicrobial activity, 3- hydroxyanthranilic acid that increase the bacterial resistance to oxidative stress during aging, and trehalose which is a carbon source for the bacteria supporting its growth.

## Discussion

*S. agalactiae* is an important host-generalist opportunistic pathogen causing disease in humans and cattle^14^. It is a leading cause of meningitis and sepsis in newborns and a causative agent of invasive infections in adults, including meningitis, endocarditis, osteoarticular, and soft tissue infections^15^. GBS bovine mastitis is a major veterinary and economic issue for the dairy industry worldwide because of the treatment costs, reduced milk production, milk that must be discarded, and culling and replacement^16^. *S. agalactiae* is recognized as an emerging pathogen in adult humans throughout the world and as a reemerging pathogen in dairy cattle^17^. Recent studies have demonstrated the importance of bioinformatics techniques and algorithms for investigating pathogenesis-related proteins and identifying druggable targets and innovative treatments^18, 19^. To our knowledge, no published study has analyzed the relationship between the proteomic profiles of clinical human and animal MDR *S. agalactiae* strains and their associated metabolites. This is the first study to generate information on the proteomic and metabolomic profiles of human versus animal GBS strains. To identify proteomic biomarkers that could better characterize the pathogenesis, we studied 23 *S. agalactiae* strains of human and animal origin. Catabolic ornithine carbamoyltransferase was the only shared protein identified by Swiss-prot between human and animal strains which is involved in arginine metabolic process. Out of the 29 differentially significant shared proteins between human and animal strains (Table 1), 26 were upregulated and 3 were downregulated in human and animal strains. Ribosomal proteins (50S ribosomal protein L13, L14, 30S ribosomal protein S3, and S5), Asp23/Gls24 family envelope stress response protein, heat shock protein 70, ornithine carbamoyltransferase (OTCase), phosphoglycerate kinase, ribonuclease R, enolase, and ribosome hibernation-promoting factors were the main group of proteins and significantly upregulated in human over animal strains. It has been hypothesized that some ribosomal proteins may be released to the cell surface or into the surrounding environment as a defensive mechanism in response to external challenges from the host immune system, antibiotics, and altering environmental conditions^20^. This may reflect the role of these ribosomal proteins in the adaptation of GBS to the host immune system and antimicrobial agents. Although ribosomal proteins are responsible for protein synthesis, it has been proposed that some ribosomal proteins may have additional or alternative functions, such as adaptation to stress and maintenance of optimal cellular function and homeostasis^21^. In this regard, upregulation of cytoplasmic ribosomal proteins was found in *Staphylococcus aureus* exposed to cold-stress conditions^20^ and in clinical *S. aureus* isolated from an osteomyelitis patient who was treated with gentamicin^22^. Along the same lines, 207 ribosomal proteins of the small and large ribosomal subparticles were identified from 2527 proteins in MDR *S. agalactiae* strains. Korobeinikova et al.^23^ have established that ribosomal proteins S1, S21, S22, S31e, and L25 are not found in all bacteria and therefore are not crucial in the translation apparatus.

Enolase is a shared protein between human and animal GBS. Enolase is a moonlighting protein with multiple functions in the cell, including glycolysis, tRNA confirmation, and a cell surface plasminogen receptor that can be activated to plasmin, promoting the spread of bacteria, penetration of endothelial cells, and organ invasion^24, 25^. The stress-related proteins (Asp23/Gls24 family envelope stress response protein and heat shock protein 70) that are highly expressed in human and animal strains may explain their role in the survival of GBS under extreme environmental conditions or during treatment. This finding is confirmed by previous studies showing that the stress-related proteins identified in *Lactiplantibacillus plantarum* 9010 help its survival under harsh gastrointestinal environmental conditions^26^. These proteins may contribute to antibiotic resistance associated with virulence^27, 28^.

Furthermore, it is evident that several proteins not only distinguish human and animal strains but are also unique to certain isolates. For instance, the protein alkyl hydroperoxide reductase subunit C (Peroxiredoxin; AhpC, X5K078) was found in one isolate (code No. 75) from cow mastitis (Supplementary Table 4). Similarly, a previous study demonstrated that AhpC protein, a member of the family of antioxidants that protects cells from toxic byproducts of oxidative metabolism, was found in two small colony variants of *S. aureus* isolates^22^. Moreover, the late competence protein ComGE (A0A1D0 CSP9), was found in one animal isolate (code No. 40). This may be due to the use of antibiotics and hydrogen peroxide in the farm. This assumption is further supported by the findings of Chanda et al.^29^ that the promoter of *S. aureus cspC* is strongly induced in response to exposure to antibiotics and hydrogen peroxide. In addition, *cspC* was present at a significantly higher level in methicillin-resistant *S. aureus* than in methicillin-sensitive *S. aureus*^28^. Mobile genetic elements, in particularly lysogenic prophages, play an important role in the evolution of GBS, its adaptation to host species, its high virulence, and its ability to cause invasive infections in humans^30^. Also, acquisition of prophages is associated with host adaptation of the cattle lineage^31^.

Antibiotic resistance is a global public health problem and a development threat. As bacteria develop resistance via the selection of existing mutations and acquisition of new genes at a rate that far outpaces our capacity to generate novel antimicrobials, our current therapeutic arsenal is becoming increasingly constrained^32^. β-Lactamases, especially carbapenem hydrolyzing enzymes, remain the greatest threat to the usage of β-lactam antibiotics^33^. One human strain (code No. 7) contained ꞵ-lactamase (serine hydrolase). This strain contains also a biofilm regulatory protein (lytR) that has been described as an important virulence regulator and a potential target for the design of novel antimicrobials^34^. Another human strain contains aminoglycosides-resistance bifunctional AAC/APH (A0A0U2QMQ5) protein that confers resistance to almost all clinically used aminoglycosides^35^.

ATP-binding cassette (ABC) transporters are one of the largest families of membrane proteins that include a number of therapeutically significant members that expel drugs. An ABC transporter from *S. pneumoniae* is responsible for fluoroquinolone resistance^36^. In this study, an ABC-type multidrug transport system with a permease component (A0A076Z2H7, A0A380IMI1, A0A829IDT8, A0A8G0MDZ4, and V6Z356) was identified in two animal strains (codes 75 and 172) and one human strain (code 24).

Moreover, a TetR family transcriptional regulator (TetR/AcrR family) involved in regulating the expression of the tetracycline resistance determinant encoded by *tet*A^37^ was found in one animal strain (code 105).

Recently, van Gool and Egmond^38^ discussed the multifaceted functions of FcαRI and IgA during homeostasis, infection, and chronic inflammation and their potential as novel therapeutic targets. It was reported that many clinical isolates of *S. pyogenes* and a majority (70%) of type lb human GBS expressed IgA Fc-receptors (ꞵ antigen)^39^. Here, the IgA FC receptor was present in only one animal isolate (code No. 91).

Kinases are crucial for bacterial growth, biofilm formation, and antibiotic resistance. Recent studies have reported the important role of penicillin-binding protein and serine/threonine kinase-associated (PASTA) kinases in regulating resistance to β-lactam antibiotics^40, 41^. Hence, bacterial kinases have received considerable attention as prospective targets for the discovery of novel antimicrobial agents^40^. Interestingly, animal strains uniquely contained histidine kinase (cssS, maeK, dcuS_2, and dcuS) and HPr kinase/phosphorylase (HPrK/P) proteins (isolates code no. 20 and 46), whereas penicillin-binding proteins 1 B and 2 B were unique in human strains (Supplementary Tables 3 and 4). In addition, two animal strains (code nos. 20 and 91) contain proteins similar to staphylokinase and streptokinase (sak), a plasminogen activator that forms plasmin, which digests fibrin clots, thus conferring access to deep tissues and facilitating the establishment of intramammary infections^42^. Recently, Salaikumaran et al.^43^ declared that there are unexplored rRNA methyltransferases (n = 40) and 16S rRNA methyltransferases (n = 13) that could provide a novel drug target for stopping the emergence of MDR bacteria as they simultaneously inhibit translation and eliminate the ability to methylate the substrates. The key finding of our study is the unique detection of 16S rRNA m5C967 methyltransferase (RsmB, sun), a TrmH family tRNA/rRNA methyltransferase YacO, cytosine-specific methyltransferase, 23S rRNA (guanosine (2251)-2′-O)-methyltransferase RlmB, rRNA methyltransferase, and methyltransferase (yhdJ_1) in human GBS. Animal GBS contains ribosomal RNA small subunit methyltransferase I (rsmI), putative tRNA (cytidine (34)-2′-O)-methyltransferase, cytosine-specific methyltransferase, and 5-methyltetrahydropteroyltriglutamate—homocysteine methyltransferase (met E).

Metabolomic investigations provide instantaneous snapshots of the physiological status of a pathogen, thereby delineating the dynamic metabolic profiles in response to stress factors, environmental alterations, useful biomarkers, and pathways involved in disease development^44^. It is a useful tool for detecting alterations in metabolites resulting from mastitis and antibiotic treatment^45, 46^. Among the annotated metabolites in human and animal GBS, uridine 5′-diphosphate, hypoxanthine, and guanine were prevalent and upregulated in human samples compared to animal samples, whereas phenylpyruvic acid was downregulated (Supplementary Table 7). These findings are consistent with those recently reported that phenylpyruvic acid, uridine, glycerol, the homogentisic acid: 4-hydroxyphenylpyruvic acid ratio, and the xanthine: ratio were prevalent in milk from dairy cows with subclinical GBS mastitis^45^. Wang et al.^47^ found that the key metabolites that distinguish the human and fish *S. agalactiae* strains were the reduced malic acid and elevated adenosine. Phenylalanine has been shown to be an important amino acid and the building block of catecholamines, which are neurotransmitters and compounds similar to adrenaline^48^. Tyrosine is created from phenylalanine and is a crucial amino acid in many proteins, peptides, and enkephalins. Tyrosine also serves as a building block for several hormones, including thyroxin and catecholoestrogens^49^. Differences in mastitis milk yield and quality may be partially explained by these variables^45^. Furthermore, it has been demonstrated that the production of glyceric acid from glycerol oxidation is mediated by the conversion of glucose carbon to serine in cows^50^. The lactating animal may develop D-glyceric aciduria and D-glycerate anaemia due to excessive glyceric acid secretion, which can lead to metabolic acidosis, progressive neurological dysfunction, seizures, and hypotonia^51^. Interestingly, the lactic acid level was upregulated in human samples. A previous study reported an increased concentration of lactic acid in milk samples from animals with subclinical and clinical mastitis and explained this increase by leukocyte metabolism. Also, they declared that concentration of the lactic acid increased significantly within 24 h after infection^52^. Therefore, lactic acid may be a viable candidate for the diagnosis of GBS mastitis in dairy cows^44^. Contrary to Thomas’ research findings^53^, the metabolic level of succinic acid increased (Supplementary Table 7). Succinic acid is an essential TCA cycle metabolite, as well as a vital precursor and intermediate product^54^. If there is a problem with this mechanism, TCA will have a similar effect on sugar, lipids, and amino acids metabolic pathways. Although succinic acid metabolism in cow mastitis is rarely documented, learning more about it would help us better understand how GBS mastitis and the TCA cycle are related and solve the incidence of dairy cow mastitis^44^. ꞵ-hydroxybutyric acid, indoleacetic acid, and phenylacetic acid were uniquely annotated in animal GBS. Moreover, butyric acid, fumaric acid, isoleucine, leucine, and hippuric acid were found in human and animal GBS. This agrees with the recent reports that specific *S. agalactiae*-related metabolites in milk can indirectly reflect whether cows have mastitis or not^44, 45^. The contents of butyric acid, β-hydroxybutyric acid, hippuric acid, fumaric acid, isoleucine, glycerol, peptides, and acetic acid increased significantly in mastitis milk with high somatic cell counts, so they can be used as new markers in mastitis milk^55, 56^.

Beside the potential biomarkers for diagnosing GBS infection, an integrated analysis of the key metabolic pathways producing the 241annotated metabolites showed that amino acid metabolism is the most important pathway, as L-aspartic acid, succinic acid, L-glutamic acid, and L-asparagine are upregulated in human strains while fumaric acid, and oxoglutaric acid are downregulated. However, Tong et al.^45^ reported carbohydrate metabolism as the most important pathway associated with GBS mastitis and downregulation of L-aspartic acid, succinic acid, L-glutamic acid, L-asparagine, fumaric acid and oxoglutaric acid. Aspartate functions in a number of physiological signalling pathways, including gluconeogenesis, and is a precursor for the biosynthesis of other important compounds^57^. In this study, alanine, aspartate, and glutamate metabolism were significantly increased (Supplementary Fig. 8). On the contrary, a previous study has reported that alanine, aspartate, and glutamate metabolism and arginine biosynthesis were significantly decreased in GBS causing subclinical mastitis in cows^45^. Pyrophosphate is a high-energy phosphate precursor that plays a significant role in monitoring energy in cells^58^. We found that isopentenyl pyrophosphate is downregulated, and farnesyl pyrophosphate is upregulated in human compared to animal strains. However, pyrophosphate levels were significantly lower in the milk of cows with GBS^45^.

Metabolome difference between human and animal strains showed 3 unique metabolites in human; L-histidine, guanosine diphosphate, and HPPA. Mainly, these metabolites are known to play crucial roles in pathogenesis, nutrient acquisition, growth, and survival of the bacteria. The role of L-histidine metabolites in *S. agalactiae* was not explicitly mentioned in the previous research. However, some relevant information about histidine metabolism in other bacteria and its potential impact on pathogenesis can be inferred. For example, *Acinetobacter baumannii* have been shown to use histidine catabolism to liberate zinc from histidine-zinc complexes during nutrient limitation^59^. Overall, while the specific role of L-histidine metabolites in *S. agalactiae* is not clear, it is possible that histidine and its metabolism play a role in the pathogenesis and nutrient acquisition of this bacterium. Further research is needed to fully understand the significance of L-histidine metabolites in *S. agalactiae*. Guanosine diphosphate metabolites are essential components for the basic cellular functions and metabolism of *S. agalactiae*. They play a role in energy production, genetic material synthesis, protein production, and cell wall maintenance, all of which are crucial for the bacterium's growth and survival^60, 61^. Understanding these metabolic pathways is important in the context of bacterial physiology and can also be relevant for developing strategies to target and control bacterial infections. There is no data that the third unique metabolite, HPPA is directly involved in GBS metabolic pathway. HPPA is a metabolic intermediate in the degradation of tyrosine in both bacteria and humans. The role of HPPA and its metabolites in bacteria is intricately tied to their metabolic and regulatory processes, aiding in energy generation, biosynthesis, detoxification, and gene expression regulation^62^. The specific function can vary depending on the bacterial species and the environmental conditions they encounter. Further investigations are required to clearly recognize the exact role of HPPA in GBS. On the other hand, 4-hydroxybenzoic acid, galactonic acid, indole-3-propionic acid, indoleacetic acid, phenylacetic acid, and phenylglyoxal were uniquely identified in animal strains. They are implicated mainly in growth and survival, energy production, antibiotic resistance, signaling, biofilm formation, and oxidative stress^63–65^.

## Conclusion

In conclusion, our findings have shed light on the proteome profile of *S. agalactiae* and their potential involvement in colonization and pathogenesis The majority of the observed proteins are mainly involved in molecular function, cellular compartments, and biological processes. Additionally, some of these proteins exhibit specific enzymatic activities. Through metabolomics, this study revealed unique metabolites associated with either human or animal *S. agalactiae* and implied that some of these metabolites annotated are not previously studied in *S. agalactiae*. Further studies are required to fully elucidate the significance of these metabolite in *S. agalactiae*. Understanding the repertoire of *S. agalactiae’s* omics profiling is important for vaccine development and could be promising tool utilized for diagnostic biomarkers of GBS.

## Materials and methods

### Bacterial strains and growth conditions

Sixteen and seven *S. agalactiae* isolates from human and animal sources; respectively, were included in this study. Human isolates were isolated from vagina of women aged from 22 to 41 years. Animal isolates were isolated from milk samples from cow mastitis. Milk was collected without any interventions were done in the mammary gland of the cow. All of them were cultured on Edward’s agar medium (Oxoid, Hampshire, England, UK) and were identified as *S. agalactiae* by CAMP positive reaction, β-hemolysis on blood agar, and positive sodium hippurate hydrolysis test^66^. Further confirmation by PCR was performed using two sets of primers targeting the *cfb* gene which encodes the CAMP factor and the *scpB* gene that encodes C5a peptidase^67^. Reference strain: *S. agalactiae* (ATCC 12386) was involved in our study.

These strains were stored at − 80 °C in 80% double strength broth and glycerol until use. The strains were cultured before use on tryptic soya agar plates then sub-cultured on brain heart infusion broth (Oxoid, Hampshire, England, UK).

### Antimicrobial susceptibility testing

The antimicrobial susceptibilities of *S. agalactiae* strains involved in our study were tested against 15 antimicrobial discs of 7 groups using the Kirby-Bauer disk diffusion assay following the Clinical and Laboratory Standards Institute guidelines and interpretative criteria^68^. The tested antimicrobial discs (Oxoid, Cambridge, UK) were penicillin (10 U), a, amoxicillin (25 µg), cloxacillin (1 µg), amoxicillin-clavulanic acid (20 + 10 µg), cephalexin (30 µg), cephoperazone (75 µg), ceftriaxone (30 µg), cefepime (30 µg), impenem (10 µg), ciprofloxacin (5 µg), tetracycline (30 µg), erythromycin (15 µg), trimethoprime -sulphamethaxazole (23.75 + 1.25 µg), streptomycin (10 µg), and clindamycin (2 µg).

### Label- free shotgun proteomic analysis

#### Extraction and preparation of bacterial proteins

Brain heart infusion broth sub-cultures of 16 and 7 *S. agalactiae* isolates from human and animal sources; respectively, were subjected to total protein extraction. Proteins were precipitated from the collected media using four times chilled acetone. After incubation at − 80 °C for 30 min and at − 20 °C overnight, samples were centrifuged at 10,000 RPM for 30 min. Protein extract of cells was obtained by adding 50 µl lysis solution (8 M urea, 500 mM Tris HCl, pH 8.5) with Complete ultra-proteases (Roche, Mannheim). Samples were incubated at 37 °C for 1 h with occasional vortex, then centrifuged at 12,000 rpm for 20 min. Protein assays of the extracts were performed using Bicinchoninic acid (BCA) assay (Pierce, Rockford IL) at Å562 nm prior to digestion.

#### Protein tryptic digestion

Thirty µg of cell protein lysate from each sample were subjected to in solution digestion. In brief, protein pellets were re-suspended in 8 M urea lysis solution and reduced with 5 mM tris (2-carboxyethyl) phosphine (TCEP) for 30 min. Alkylation of Cysteine residues was performed using 10 mM iodoacetamide for 30 min in dark area. Samples were diluted to final concentration 2 M urea with 100 mM Tris-HCl, pH 8.5 prior to digestion with trypsin. For endopeptidase digestion, modified porcine trypsin (Sigma, Germany) was added at 40:1 (protein: protease mass ratio) and incubated overnight in a thermo-shaker at 600 rpm at 37 °C. Digested peptide solution was acidified using 90% formic acid to a final pH of 2.0. The resultant peptide mixture was cleaned up using stage tip as discussed earlier^69^. Peptides were assayed using BCA method (Pierce, Rockford IL) at Å562 nm prior to injection to be 1.5ug/10 µl.

#### Nano-LC MS/MS analysis

Nano-LC MS/MS analysis was performed using TripleTOF 5600 + (AB Sciex, Canada) interfaced at the front end with Eksigent nanoLC 400 autosampler with Ekspert nanoLC 425 pump. Peptides were trapped on CHROMXP C18CL 5 µm (10 × 0.5 mm) (Sciex, Germany) on trap and elute mode. MS and MS/MS ranges were 400–1250 m/z and 170–1500 m/z, respectively. A design of a 120-min liner gradient 3–80% solution (80% ACN, 0.2% formic acid) was performed. The 40 most intense ions were sequentially selected under data dependent acquisition (DDA) mode with a charge state 2–5. For each cycle, survey full scan MS and MS/MS spectra were acquired at resolution of 35.000 and 15.000, respectively. For high precision, external calibration was programmed and run during sample batches to correct for possible TOF deviation.

#### Proteomics data processing

Raw LC/MS/MS data in Wiff format were searched using ProteinPilot™ Software^70^. Peptides were identified from MS/MS spectra, then the Pro Group™ Algorithm was used to assemble peptide identifications into a list of protein identifications. *S. agalactiae* database (Swissport and TrEMBL database containing 24,299 entries, organism ID 1311, download date: 7.3.2022) was used. Analysis was searched with Bias Correction using and biological modifications as ID focus. To ensure high-quality results, the false discovery rate (FDR) was maintained at 1% of the protein level. All figures are generated using the ggplot 2 package for the statistical programming language R^71^.

### Metabolomic analysis

#### Extraction and preparation of bacterial metabolites

Brain heart infusion broth (BHI) sub-cultures of 16 and 7 *S. agalactiae* isolates from human and animal sources; respectively, were subjected to total metabolites extraction. Both intra- and extra-cellular metabolites were used by vertexing the bacterial pellets in the BHI broth. One mL of each broth culture was thawed on ice. A pre-cooled methanol 80% was added to each sample^72^. A 100 µl of methanol containing 10 ng/mL internal standards (Olopatadine and Atorvastatin) was added to each sample then agitation for 2 min followed by thawing and freezing for two cycles, 15 min each. The mixture was vortexed and whirled for at least 2 min. Samples were ultra-sonicated for 15 min at a temperature less than 25 °C and were centrifuged at 12,000 rpm at 4 °C for 10 min. The supernatant was transferred to a clean tube. And each sample was further dried using a speed vac at 30 °C and reconstituted in a solvent consisting of water: methanol: acetonitrile 2:1:1, respectively. The extracted samples were then subjected to LC–MS/MS analysis. A pooled quality control (QC) was obtained by mixing 10 µl of each sample.

#### Liquid chromatography mass spectrometic (LC MS/MS) analysis

Chromatographic separation was carried out using a SCIEX EXION LC™ AC UHPLC system equipped with Acquity XSelect HSS T3 analytical column 2.1 × 150 mm, 2.5 µm ID (Waters Co, Milford, US). Mass spectrometric analysis was performed using Triple TOF™ 5600 + system (AB SCIEX, Concord, Canada) equipped with a Duo-Spray source operated using the positive-ion (ESI^+^) and negative-ion (ESI^–^) modes. IDA acquisition method consisted of a single TOF scan from 50 to 1100 Da followed by product ion TOF scan from 50 to 1000. The mobile phase solutions consisted of solution (A); 5 mM ammonium formate in 1% methanol (pH 3.0) for positive mode, solution (B); acetonitrile, and solution (C); 5 mM ammonium formate in 1% methanol (pH 8.0) for negative mode. Gradient elution was sustained at 0% B for 1.0 min, 0% to 90% B in 20 min, 90% for 4.0 min, 90% B to 0% B in 1.0 min, and finally re-equilibrating with 0% B for 3.0 min. The order for samples was random, with an injection of QC sample every ten injections. Mass calibration was automatically performed every 2.5 h analysis by an automated calibration delivery system using APCI calibration solution (AB SCIEX). Blank sample used to check solvents quality and monitor carryover. Data collections were created using Analyst TF (v 1.7.1).

#### Metabolomics data processing

MS-DIAL platform was used for small molecules identification^73^. High-resolution microbial database collected from HMDB workbench was used as a search space. Precursor ion XIC signal in sample: blank > 5 with a precursor mass tolerance of 10 ppm was applied. The detected and identified small molecules were used for subsequent analysis. To give more credence for identified molecules, manual validation was performed to confirm ideal alignment using PeakView 2.2 with MasterView 1.1 (AB SCIEX) to validate the MS1 and MS2 of the significant molecules.

### Bioinformatic and statistical analysis

Protein abundance represented by NSAF was subjected to filtration process based on its appearance within the replicates where proteins were omitted if it did not show within 20% group. Data were normalized using Probabilistic Quotient Normalization (PQN)^74^, Log transformed and scaled. All process was achieved using R environment. Differential expression analysis was performed using Mann Whitney test with FDR 1%^75^. For metabolomics, peak intensities were parsed for further analysis. Filtration process was applied with a cutoff 50% group and FDR < 0.1 (10%). Data were normalized using PQN and scaled then subjected to statistical analysis. For metabolomics, T test was used. Heatmap analysis was plotted based on NSAF abundance after applying the abovementioned parameters with *p* value 0.05 and FDR 1%. Dendrogram associated with heatmap was generated using the Euclidean distance calculation. KEGG pathway maps were created using the KEGG database^76^.

### Ethical approval

This study was approved by Suez Canal University ethical board (No. 202103RH2). All experiments were performed in accordance with relevant guidelines and regulations. An informed consent was obtained from all subjects in this study.

### Supplementary Information

Supplementary Legends.

Supplementary Figure 1.

Supplementary Figure 2.

Supplementary Figures.

Supplementary Figure 3.

Supplementary Figure 4.

Supplementary Figure 5.

Supplementary Figure 6.

Supplementary Figure 7.

Supplementary Figure 8.

Supplementary Table 1.

Supplementary Table 2.

Supplementary Table 3.

Supplementary Table 4.

Supplementary Table 5.

Supplementary Table 6.

Supplementary Table 7.

## Data Availability

All data generated and analyzed during this study are included in this article and published online on PRIDE database (www.ebi.ac.uk/pride) via ProteomeXchange with identifier PXD035678.
