# Supplementary material for: Proteomics and metabolomics analyses of Streptococcus agalactiae isolates from human and animal sources
Source: Sci Rep. 2023 Nov 28;13:20980. doi: 10.1038/s41598-023-47976-y (PMC10684508; doi:10.1038/s41598-023-47976-y)
Supplement: Supplementary file 1 — Supplementary Legends. [file 41598_2023_47976_MOESM1_ESM.docx]

**Supplementary data Legends**

**Supplementary Table 1:** Resistance phenotype of 23 *S. agalactiae* isolates recovered from pregnant women and cow mastitis

**Supplementary Table 2:** All identified proteins in human and animals *S. agalactiae* strains

**Supplementary Table 3:** Uniquely identified proteins in human *S. agalactiae* strains

**Supplementary Table 4:** Uniquely identified proteins in animals *S. agalactiae* strains

**Supplementary Table 5:** Gene ontology annotation for proteins of *S. agalactiae* strains of human origin

**Supplementary Table 6:** Gene ontology annotation for proteins of *S. agalactiae* strains of animal origin

**Supplementary Table 7:** All annotated metabolites in human and animals *S. agalactiae* strains; Raw metabolites (A), After filtration (B), and Annotated metabolites.

**Supplementary Figure 1:** Kernel density plots and box plots before and after normalization

**Supplementary Figure 2:** Gene Ontology annotation for proteins of *S. agalactiae* strains of human (A) and animal (B) origin

**Supplementary Figure 3:** KEGG orthology annotation for proteins in human *S. agalactiae* strains

**Supplementary Figure 4:** KEGG orthology annotation for proteins in animals *S. agalactiae* strains

**Supplementary Figure 5:** Fold change of shared proteins in human and animals *S. agalactiae* strains

**Supplementary Figure 6:** PCA (A) and OPLS-DA (B) plots of metabolites in human and animal *S. agalactiae* strains

**Supplementary Figure 7:** Orthogonal partial least-squares-discriminant analysis (OPLS-DA) validation plot of metabolites in human and animal *S. agalactiae* strains

**Supplementary Figure 8:** The shared metabolic pathways between human and animal *S. agalactiae* strains.
