# Supplementary figures and images for: Proteomics and metabolomics analyses of Streptococcus agalactiae isolates from human and animal sources
Source: Sci Rep. 2023 Nov 28;13:20980. doi: 10.1038/s41598-023-47976-y (PMC10684508; doi:10.1038/s41598-023-47976-y)

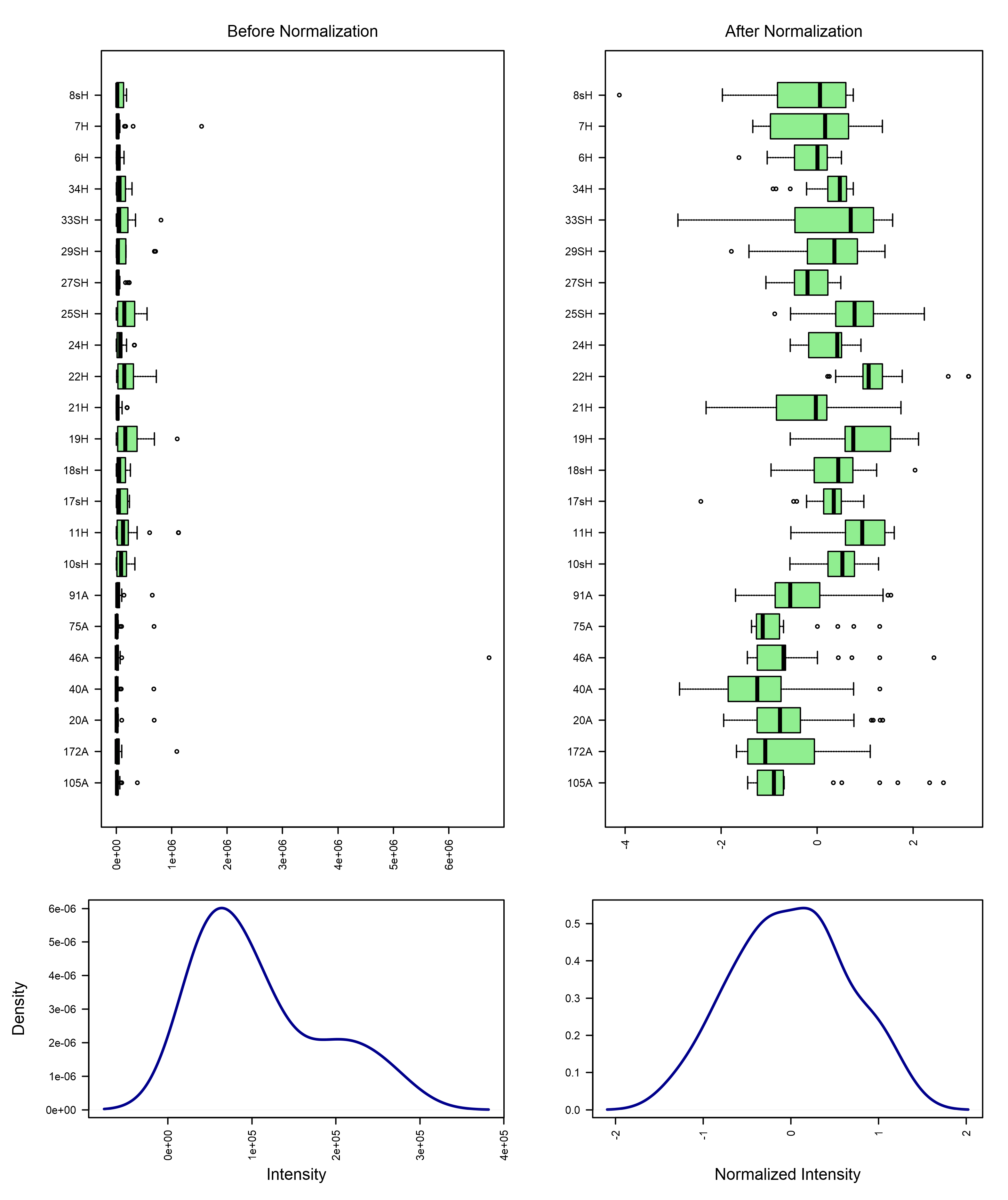

Supplement: Supplementary file 2 — Supplementary Figure 1. [file 41598_2023_47976_MOESM2_ESM.png]

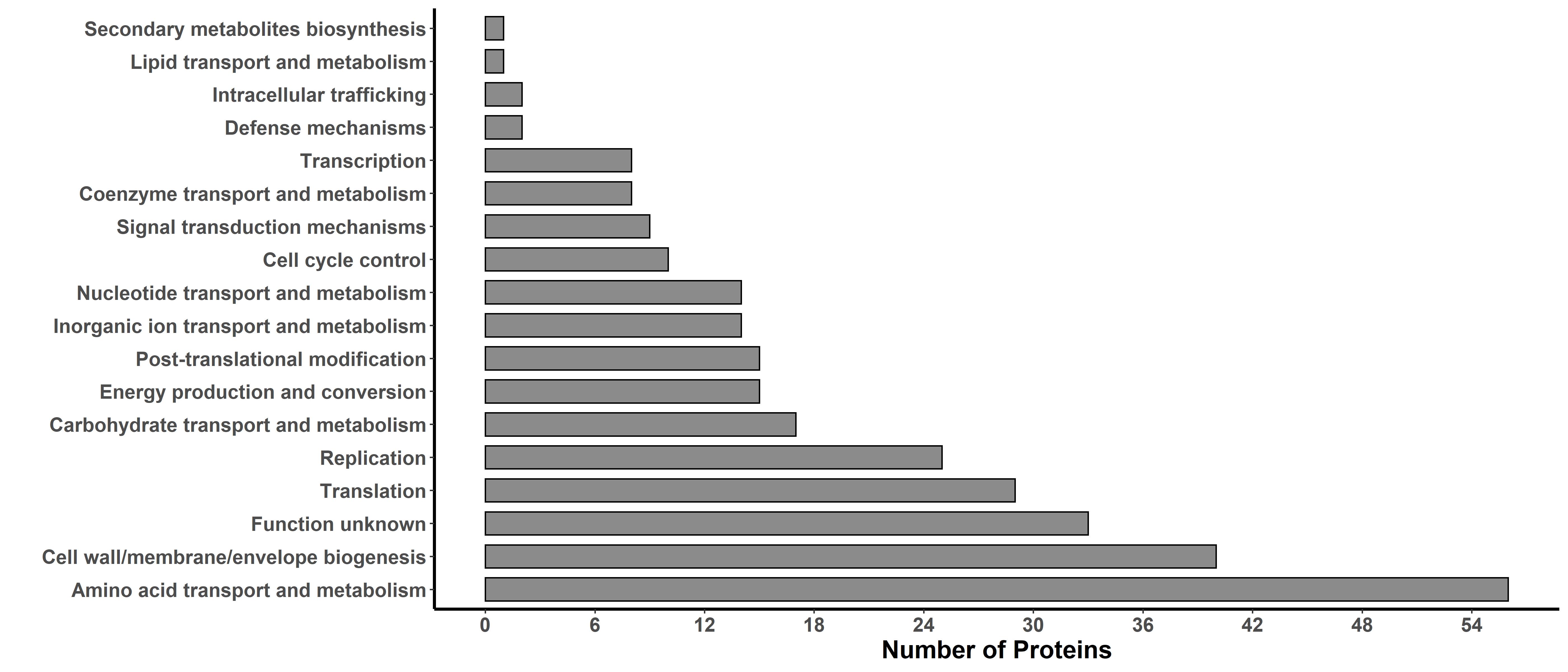

Supplement: Supplementary file 3 — Supplementary Figure 2. [file 41598_2023_47976_MOESM3_ESM.jpg]

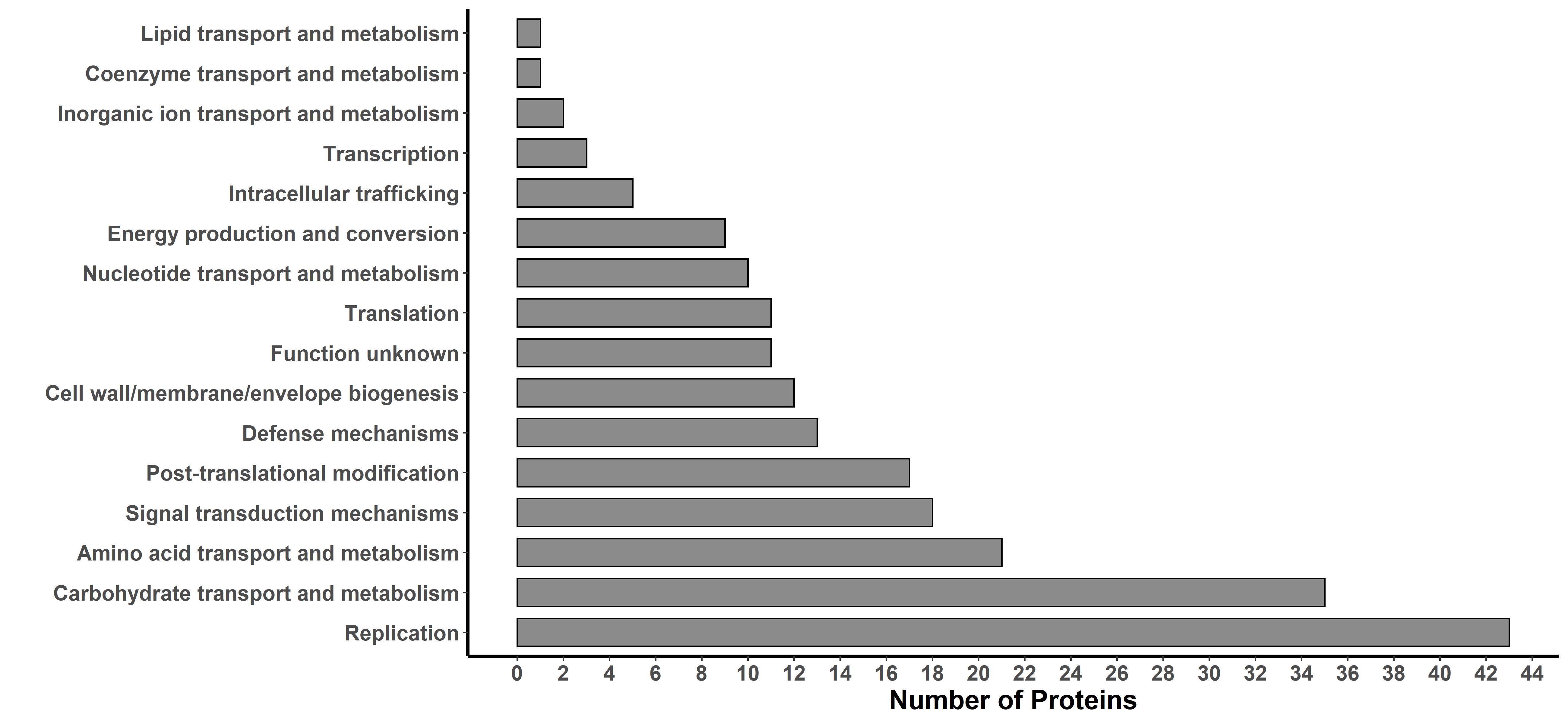

Supplement: Supplementary file 4 — Supplementary Figures. [file 41598_2023_47976_MOESM4_ESM.jpg]

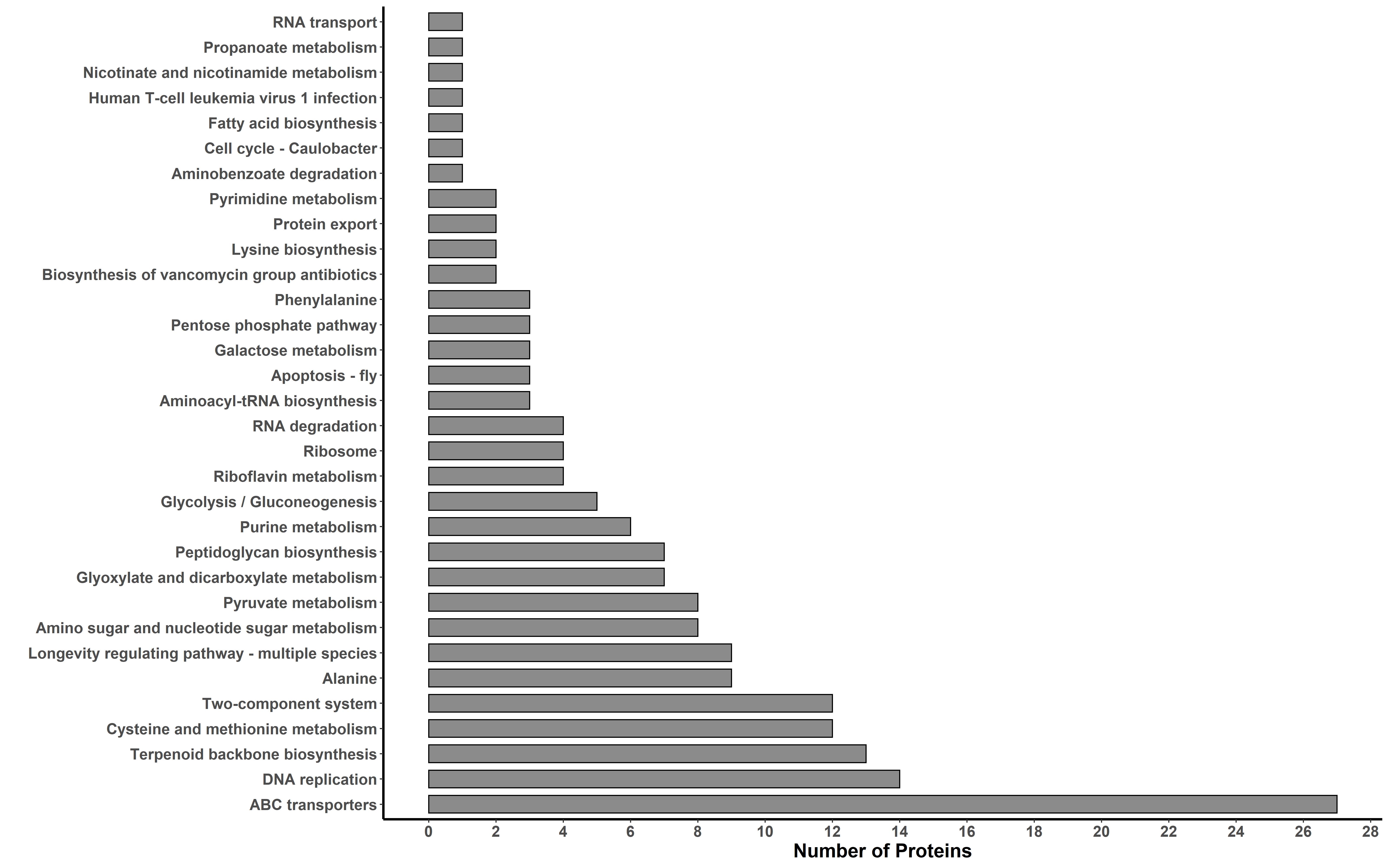

Supplement: Supplementary file 5 — Supplementary Figure 3. [file 41598_2023_47976_MOESM5_ESM.jpg]

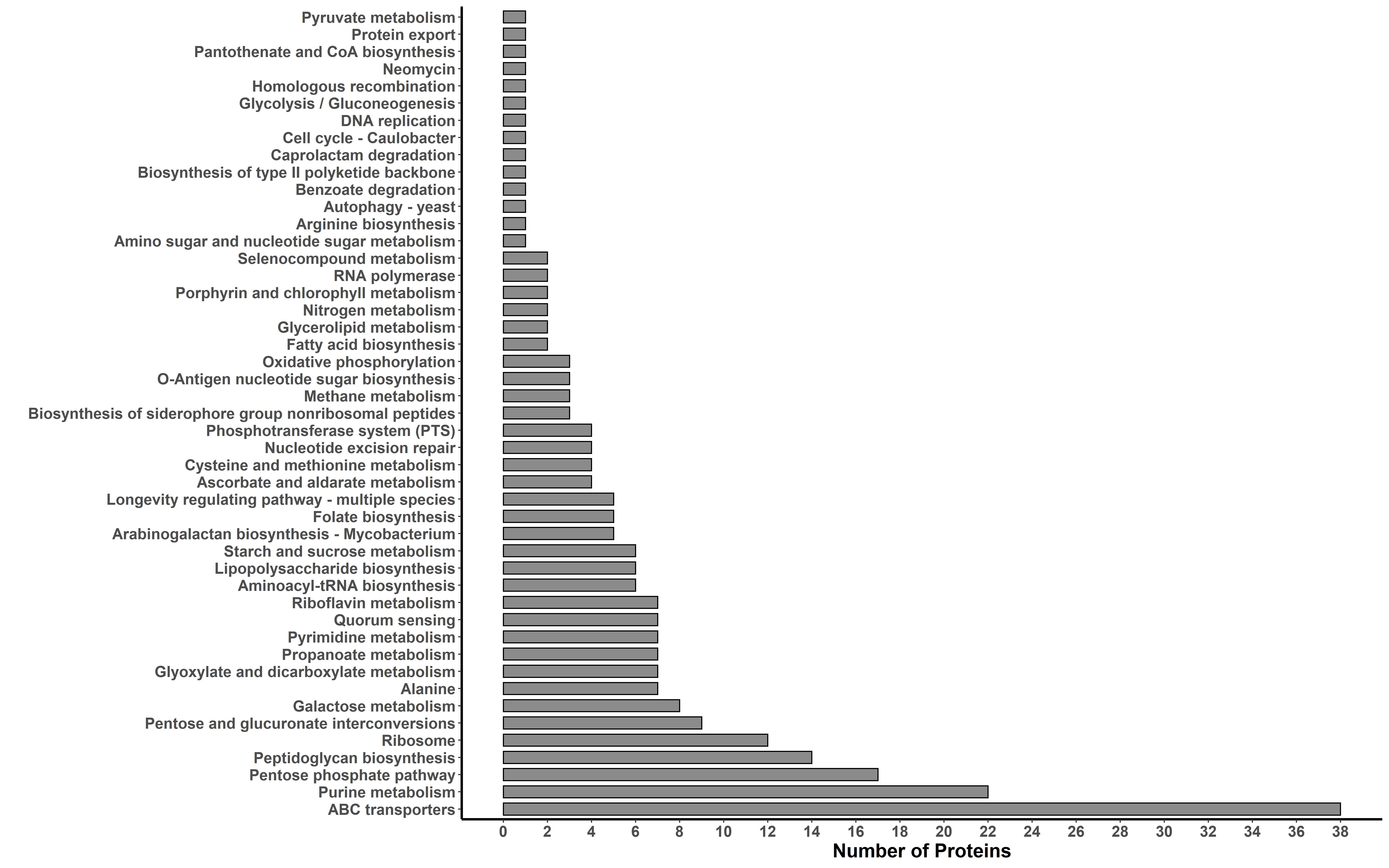

Supplement: Supplementary file 6 — Supplementary Figure 4. [file 41598_2023_47976_MOESM6_ESM.jpg]

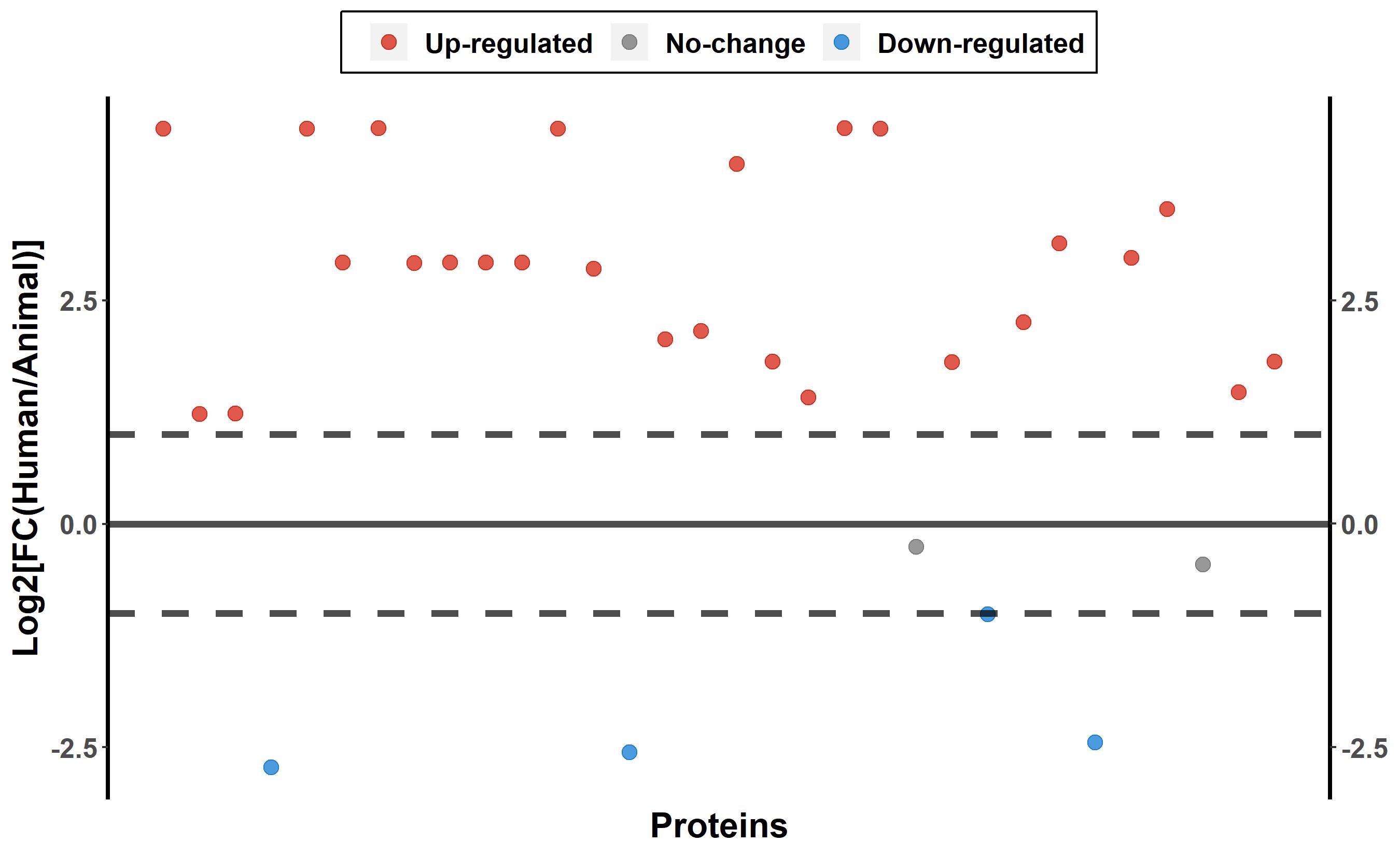

Supplement: Supplementary file 7 — Supplementary Figure 5. [file 41598_2023_47976_MOESM7_ESM.jpeg]

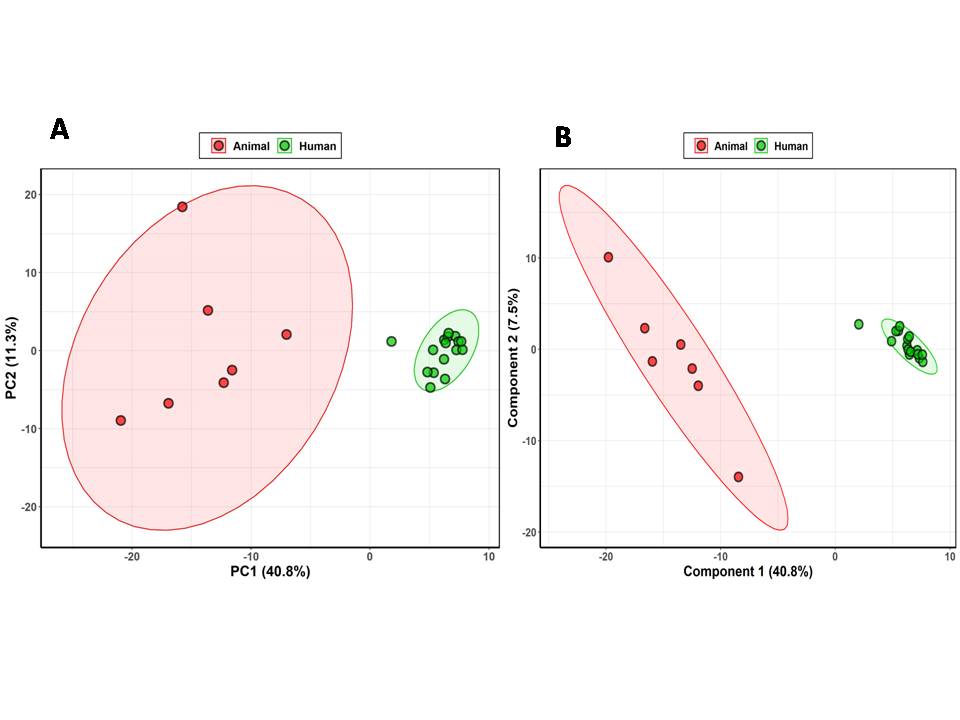

Supplement: Supplementary file 8 — Supplementary Figure 6. [file 41598_2023_47976_MOESM8_ESM.jpg]

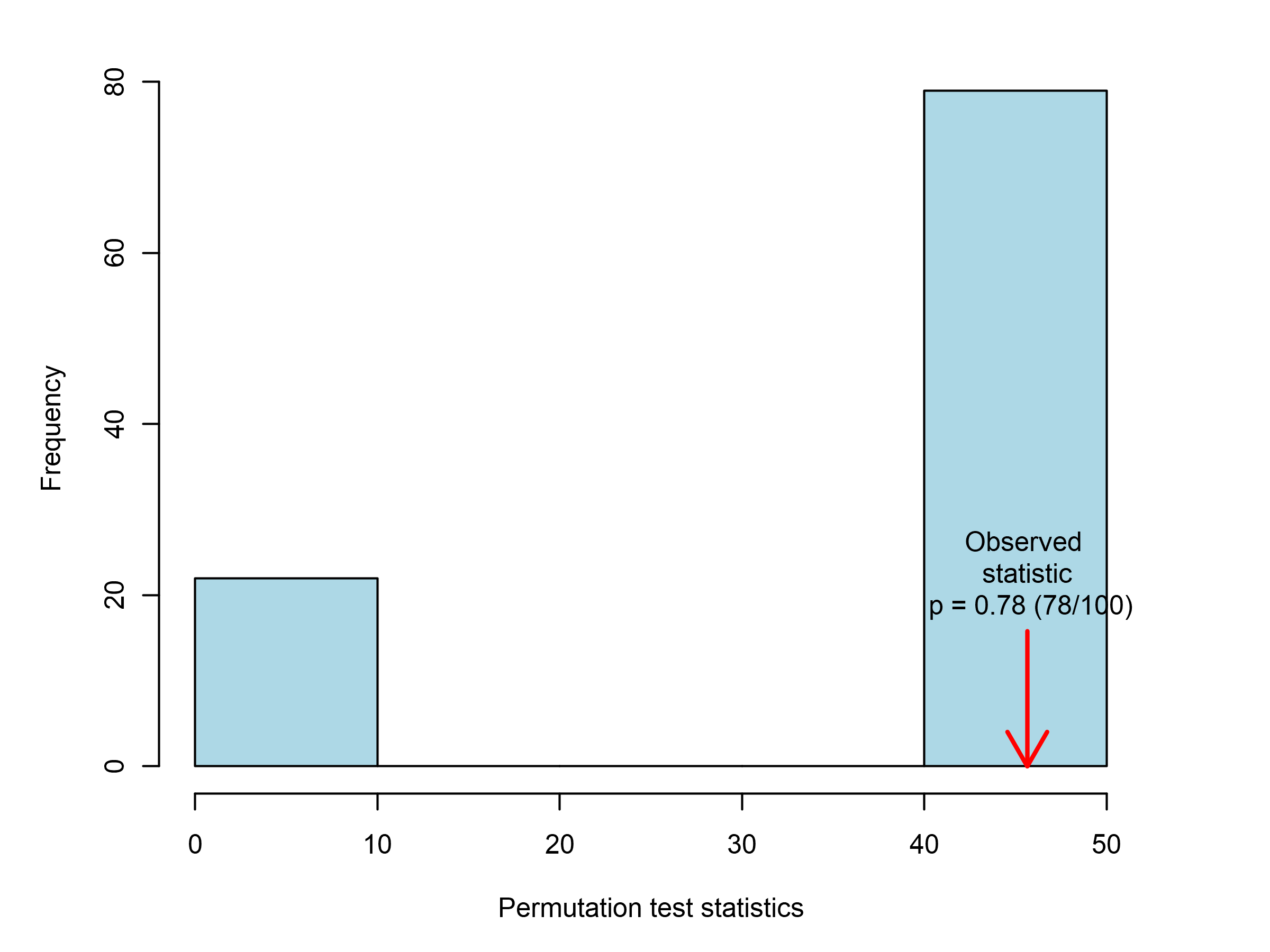

Supplement: Supplementary file 9 — Supplementary Figure 7. [file 41598_2023_47976_MOESM9_ESM.png]

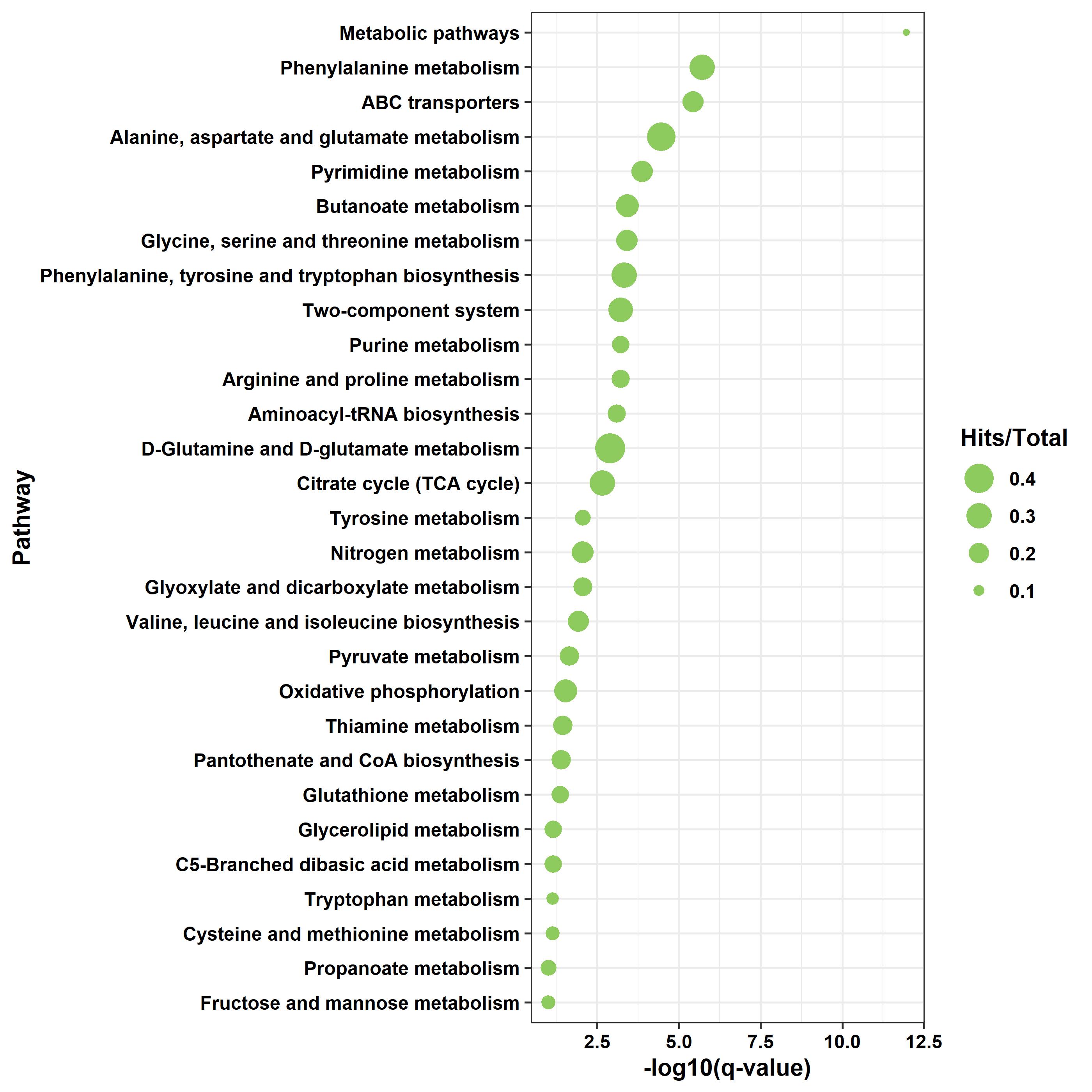

Supplement: Supplementary file 10 — Supplementary Figure 8. [file 41598_2023_47976_MOESM10_ESM.jpg]
