# Supplementary material for: Proteomics and metabolomics analyses of Streptococcus agalactiae isolates from human and animal sources
Source: Sci Rep. 2023 Nov 28;13:20980. doi: 10.1038/s41598-023-47976-y (PMC10684508; doi:10.1038/s41598-023-47976-y)
Supplement: Supplementary file 11 — Supplementary Table 1. [file 41598_2023_47976_MOESM11_ESM.doc]

**Supplementary Table 1: Resistance phenotype of 23 *S. agalactiae* isolates recovered from pregnant women and cow mastitis**

| **Isolate code No.** | **Source** | **P** | **AX** | **S** | **AMC** | **CIP** | **CX** | **IMP** | **TE** | **E** | **CEP** | **CL** | **FEP** | **DA** | **SXT** | **CRO** | **MAR index** |
| --- | --- | --- | --- | --- | --- | --- | --- | --- | --- | --- | --- | --- | --- | --- | --- | --- | --- |
| 6 H | 37 years old woman | R | R | R | S | R | R | S | R | R | R | R | R | R | R | R | 0.86 |
| 7 H | 25 years old woman | R | R | R | R | R | R | S | R | R | R | R | R | R | S | R | 0.86 |
| 8s H | 24 years old woman | R | R | R | I | R | R | S | R | R | S | R | R | R | S | R | 0.73 |
| 10s H | 32 years old woman | R | R | R | R | R | R | S | R | R | I | R | R | R | S | R | 0.8 |
| 11 H | 30 years old woman | R | R | R | R | R | R | S | R | R | I | R | R | R | S | R | 0.8 |
| 17s H | 41 years old woman | R | R | R | I | R | R | S | R | R | R | R | R | R | R | R | 0.86 |
| 18s H | 29 years old woman | R | R | R | R | R | R | S | R | R | R | R | R | R | S | R | 0.86 |
| 19 H | 31 years old woman | R | R | R | R | R | R | S | R | R | I | R | R | R | S | R | 0.8 |
| 21 H | 28 years old woman | R | R | R | I | R | R | S | R | R | S | R | R | R | S | R | 0.73 |
| 22 H | 35 years old woman | R | R | R | R | R | R | S | R | R | R | R | R | R | R | R | 0.93 |
| 24 H | 33 years old woman | R | R | R | R | R | R | S | R | R | I | R | R | R | R | R | 0.86 |
| 25s H | 27 years old woman | R | R | R | R | R | R | S | R | R | R | R | R | R | S | R | 0.86 |
| 27s H | 26 years old woman | R | R | R | S | R | R | S | R | R | S | R | R | R | S | R | 0.73 |
| 29s H | 22 years old woman | R | R | R | I | R | R | S | R | R | I | R | R | R | S | R | 0.73 |
| 33s H | 37 years old woman | R | R | R | R | R | R | S | R | R | R | R | R | R | R | R | 0.93 |
| 34 H | 25 years old woman | R | R | R | R | R | R | S | R | R | I | R | R | R | S | R | 0.8 |
| 20 A | Cow mastitis, Al-Alamia farm, Giza Governorate | R | R | R | S | I | R | S | R | R | S | R | R | R | S | R | 0.66 |
| 40 A | Cow mastitis, Saeid Elalfy farm, Alexandria Governorate | R | R | R | R | I | R | S | R | R | I | R | R | R | S | R | 0.73 |
| 46 A | Cow mastitis, Saeid Elalfy farm, Alexandria Governorate | R | R | R | S | S | R | S | R | R | I | R | R | R | S | R | 0.66 |
| 75 A | Cow mastitis, Dina farm, Alexandria Governorate | R | R | R | R | I | R | S | R | R | R | R | R | R | R | R | 0.86 |
| 91 A | Cow mastitis, Al-Haraky Farm Sharkia Governorate | R | R | R | S | I | R | S | R | R | R | R | R | R | R | R | 0.8 |
| 105 A | Cow mastitis, Al-Haraky Farm, Sharkia Governorate | R | R | R | R | S | R | S | R | R | I | R | R | R | S | R | 0.73 |
| 172 A | Cow mastitis, Sporadic case from Belbies, Sharkia Governorate | R | R | R | R | S | R | S | R | R | R | R | R | R | I | R | 0.8 |
| **Total resistant isolates (%)** |  | 23 (100) | 23 (100) | 23 (100) | 14  (60.9) | 16  (69.6) | 23 (100) | 0  (0.00) | 23 (100) | 23 (100) | 10  (43.5) | 23 (100) | 23 (100) | 23 (100) | 7  (30.4) | 23 (100) | NE |

P: Penicillin, AX: Amoxicillin, S: Streptomycin, AMC: Amoxicillin- clavulanic acid, CIP: Ciprofloxacin, CX: Cloxicillin, IMP: Impenem, CRO: Ceftriaxone, TE: Tetracycline, E: Erythromycin, CEP: Cefoperazone, CL: Cephalexin, FEB: Cefepime, DA: Clindamycin, SXT: Timethoprime- sulfamethaxole. MAR: multiple antibiotic resistance, S: Sensitive, I: Intermediate, R: Resistant, NE: not estimated
